# Harmonic radar tracking reveals that honeybee drones navigate between multiple aerial leks

**DOI:** 10.1016/j.isci.2021.102499

**Published:** 2021-05-20

**Authors:** Joseph L. Woodgate, James C. Makinson, Natacha Rossi, Ka S. Lim, Andrew M. Reynolds, Christopher J. Rawlings, Lars Chittka

**Affiliations:** 1 School of Biological and Chemical Sciences, Queen Mary University of London, London E1 4NS, UK; 2 Department of Computational and Analytical Sciences, Rothamsted Research, Harpenden AL5 2JQ, UK

**Keywords:** Environmental science, Ecology, Biological sciences, Zoology, Animals, Ethology

## Abstract

Male honeybees (drones) are thought to congregate in large numbers in particular “drone congregation areas” to mate. We used harmonic radar to record the flight paths of individual drones and found that drones favored certain locations within the landscape which were stable over two years. Drones often visit multiple potential lekking sites within a single flight and take shared flight paths between them. Flights between such sites are relatively straight and begin as early as the drone's second flight, indicating familiarity with the sites acquired during initial learning flights. Arriving at congregation areas, drones display convoluted, looping flight patterns. We found a correlation between a drone's distance from the center of each area and its acceleration toward the center, a signature of collective behavior leading to congregation in these areas. Our study reveals the behavior of individual drones as they navigate between and within multiple aerial leks.

## Highlights

• Flight paths of individual honeybee drones were tracked using harmonic radar
• Convoluted flights were concentrated in four drone congregation areas
• Drones commonly move between lek-like congregation areas during a single flight
• Acceleration patterns suggest a mechanism to maintain congregation area cohesion

## Graphical abstract

**Figure:**
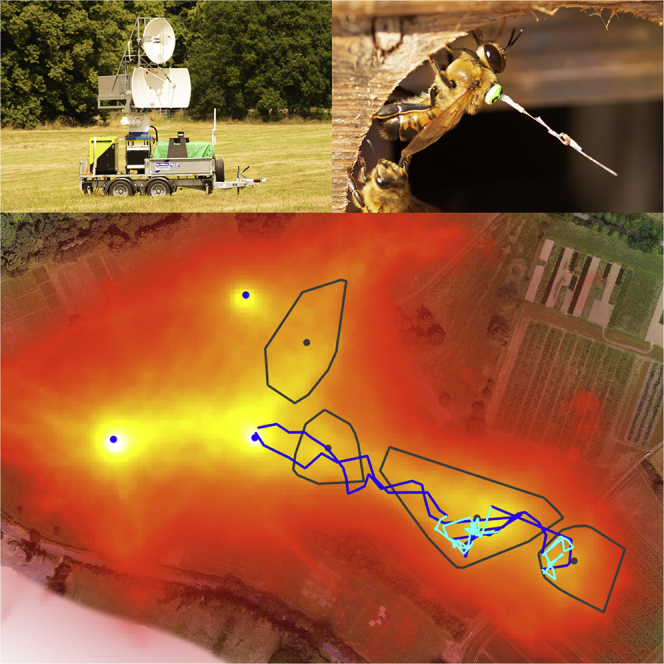

## Introduction

A mystery regarding honeybee (*Apis mellifera*) mating behavior concerns where mating takes place and how drones (males) and queens find one another. Drones attempt to mate with virgin queens in flight and typically undertake 1–6 flights per day (Witherell, 1971; Reyes et al., 2019), over an average of 7 non-consecutive days (Reyes et al., 2019), until they mate successfully or die of predation or old age (mean age at death: 21 days [Witherell, 1971; Reyes et al., 2019]). A long-standing hypothesis suggests that drones gather in large numbers, up to many thousands at a time (Koeniger et al., 2005a), in locations that are not only stable from day to day but also reappear in the same places year after year (Ruttner and Ruttner, 1966; Strang, 1970; Loper et al., 1992). Support for this drone congregation area hypothesis comes from studies using tethered queens or pheromone lures to sample drone abundance (Zmarlicki and Morse, 1963; Ruttner and Ruttner, 1972; Taylor, 1984; Galindo-Cardona et al., 2012), but there is limited evidence that such gatherings occur in the absence of the methods used to detect them (Loper, Wolf and Taylor, 1987, 1992), and other lure studies have yielded contradictory evidence (Butler and Fairey, 1964; Currie, 1987).

Nearly all investigations of drone congregations have relied on pheromone lures or tethered queens, leading to concerns that apparent congregation areas may have been created by the lures themselves. Apparent congregations can be created by releasing large amounts of pheromone (Butler, 1967; Strang, 1970; Tribe, 1982), and drones return frequently to locations at which they have encountered queen pheromone (Butler and Fairey, 1964), so such artificial congregations may be long lasting. Several authors report that drones were rapidly attracted to pheromone lures in almost any location (Butler and Fairey, 1964; Tribe, 1982), including 800 m out to sea (Butler and Fairey, 1964), leading Butler and Fairey to conclude that drones must be dispersed widely and evenly throughout the landscape (Butler and Fairey, 1964). While lure sampling studies in hilly regions have reported patterns of attraction to lures suggestive of distinct drone congregations (Ruttner, 1966; Ruttner and Ruttner, 1966), this has been hard to replicate in flatter areas (Ruttner, 1966; Currie, 1987). To demonstrate the existence of drone congregation areas with certainty, it is necessary to show that drones congregate in these areas without the presence of such bait.

Two previous studies have used radar technology to attempt to characterize the movements of drones, although they could not identify or track the flight paths of individual drones. Loper et al. (1987) used an X-band (9.4Ghz) marine radar to confirm that drones were present at purported drone congregation areas even in the absence of queens. However, since caged queens had been used to identify these locations to begin with, it was impossible to rule out the possibility that the congregations had become established as a result of the lures. In a more ambitious study, Loper and others used radar to survey the numbers of drones observed in different locations around a large apiary and built up a picture of drone movements, in the aggregate (Loper et al., 1992). They described a network of 18 km of shared flyways in which thousands of drones followed very similar routes throughout the landscape. These flyways were 50-100 m wide and often ran parallel (but no closer than 60 m) to tree lines and roadways. They identified 26 different locations they believed to be drone congregation areas (Loper et al., 1992). Congregation areas had diameters around 100 m and tended to be higher than flyways (around 30 m) but were described as an “inverted cone” in which fewer drones were found at higher altitudes (Loper et al., 1992). In a sub-experiment, Loper et al. (1992) monitored two of these purported congregation areas throughout the course of one afternoon to observe how the number of drones varied with time of day. They reported a maximum of 68 drones at a congregation at any one time, which is very low compared to the numbers found by other studies (Koeniger et al., 2005a).

Almost nothing is yet known of the flight dynamics of individual drones, how they explore the landscape, how their behavior changes at congregation areas, or whether they are faithful to a single congregation area. Among vertebrates with lek mating systems—characterized by spatial clusters of large numbers of males, who are there solely to attempt to mate and do not provide any direct benefits to females, such as food or territory (Bradbury, 1977; Alcock, 1987)—males show high levels of fidelity to a single lek (Apollonio et al., 1989; Figenschou et al., 2004; Gibson et al., 2014; Fremgen et al., 2017); it is not known whether lekking insects are similarly faithful to a single site, although there is some evidence that at least one species of wasp may be (Nielsen and Nielsen, 1953). Additionally, a body of literature on the placement and composition of congregations rests on the central assumption that the use of pheromone or queen lures does not alter drone behavior. The only support for this comes from a single radar study (Loper et al., 1992), which contradicts most other literature in suggesting that congregations are smaller, more numerous and closer together than previously thought, and which thus requires further investigation.

## Results

### Use of the landscape by drones

We tracked the flights of honeybee drones (*Apis mellifera*) from three hives in a hay meadow set within an agricultural landscape at Rothamsted Research, Hertfordshire, UK, over two years, from June to September 2016 and from May to July 2017. Drones were allowed to leave and enter the hives at will. They were tracked by harmonic radar when they chose to fly. We recorded 648 “substantial flight segments”—defined as a series of positional fixes from the radar which could be unambiguously identified as being made by a single drone, lasting at least 30 s, in which the bee moved at least 15 m from its starting position—from at least 78 individual drones.

**Figure 1:**
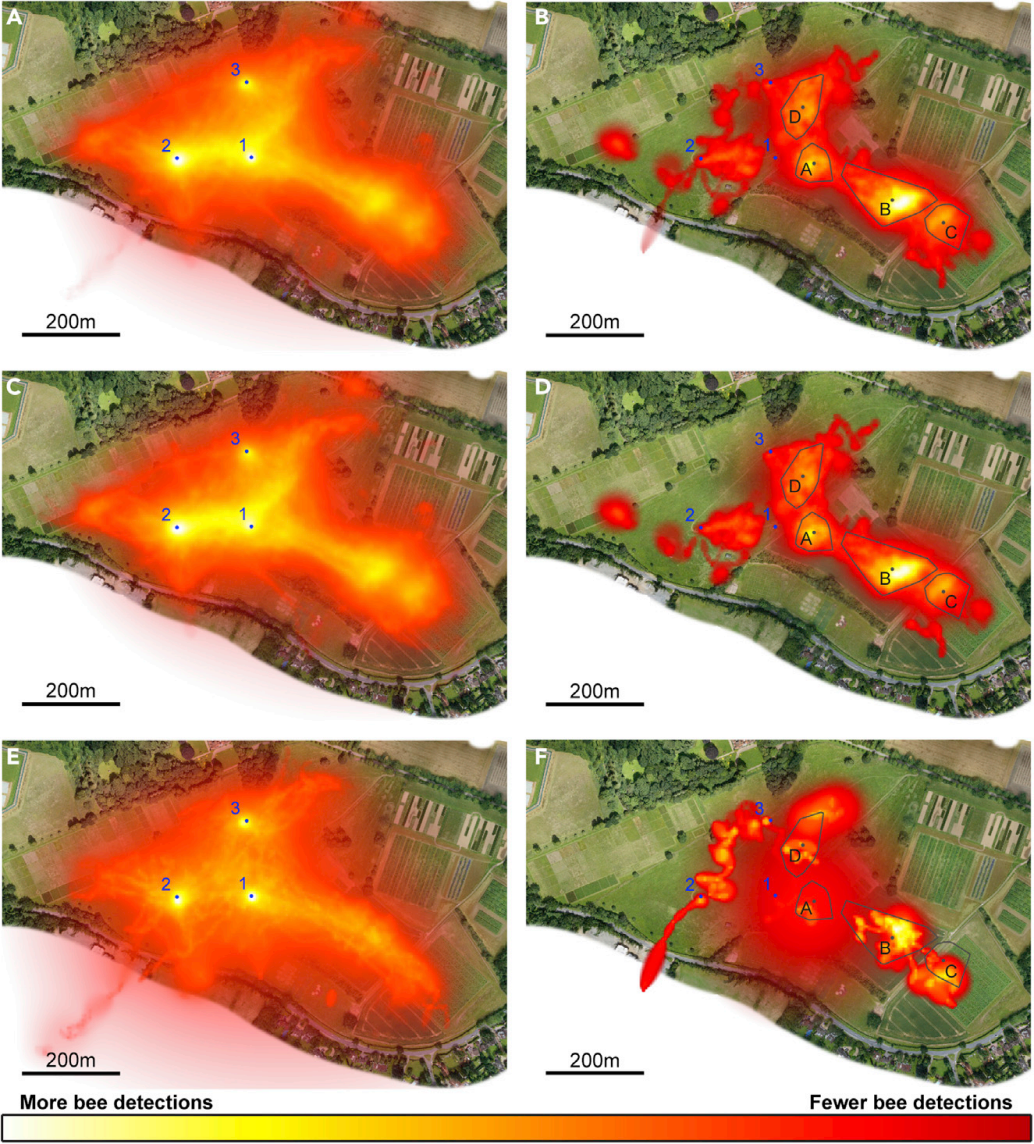
Landscape use by drones (A) Heat map showing all drone flight activity recorded in 2016-2017 superimposed on an aerial orthomosaic image of the field site. Hive locations are marked by blue circles and numbered. Areas with brighter, yellower coloration were more visited by drones. N = 1174 tracks. Scale shown by bar in bottom left corner of panel. (B) Heat map showing all convoluted sections of flight recorded in 2016-2017, whose center of mass was greater than 50 m from all active hives. The center of mass of each cluster of data points that we identified as a probable drone congregation area is marked by a gray circle and labeled A-D. Convex hull polygons containing all data points assigned to each cluster are outlined in gray. This is a rough estimate of the boundary of each congregation area, for illustrative purposes only. N = 111 tracks. (C) Heat map showing all drone activity recorded in 2016. N = 835 tracks. (D) Heat map showing convoluted sections of flight recorded in 2016, whose center of mass was greater than 50 m from all active hives. N = 94 tracks. (E) Heat map showing all drone activity recorded in 2017. N = 339 tracks. (F) Heat map showing convoluted sections of flight recorded in 2017, whose center of mass was greater than 50 m from all active hives. N = 17 tracks. See also Figures S1, S2, and S10; Table S1.

**Figure 2:**
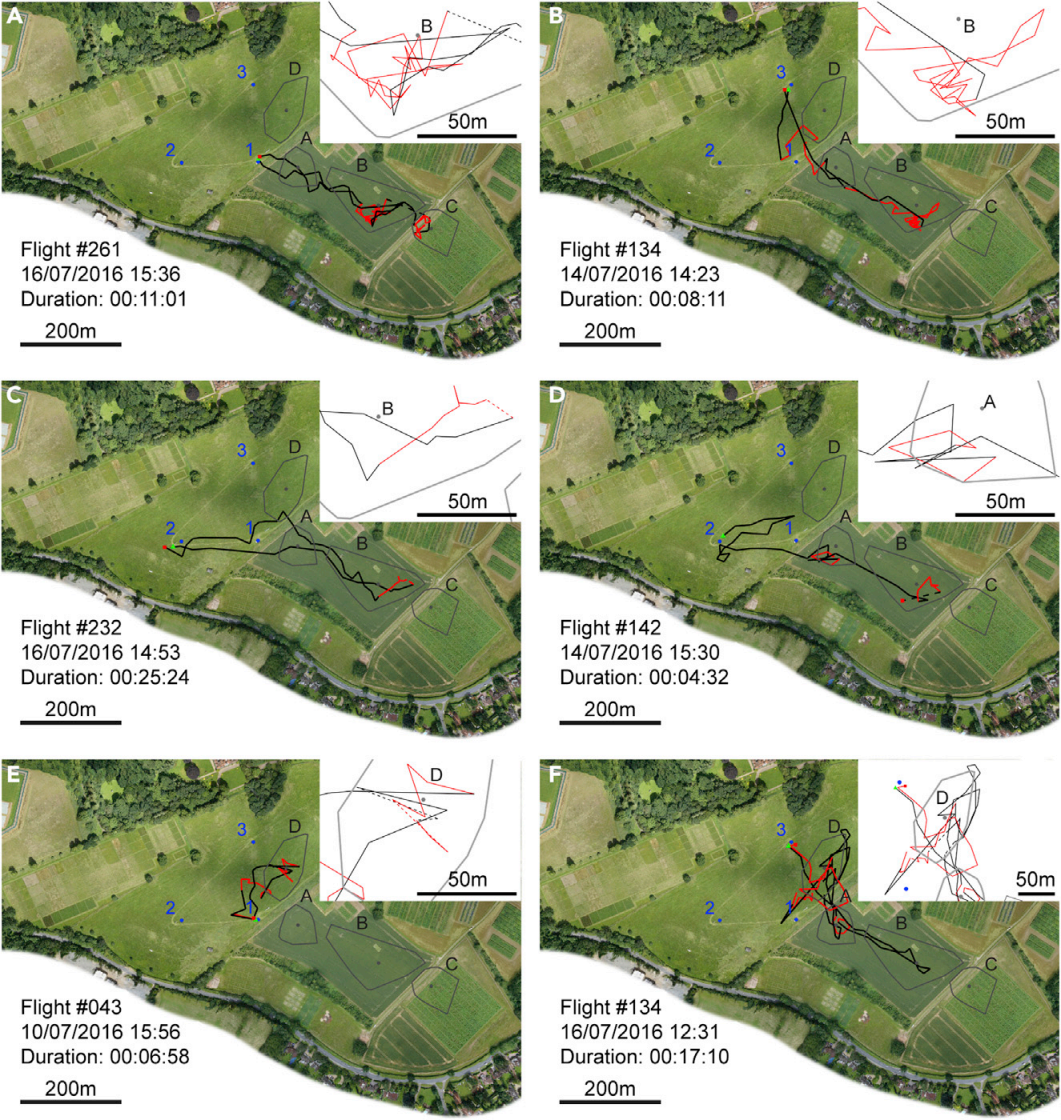
Example flight paths showing convergence on similar routes and visits to multiple congregation areas (A) Flight path of a drone from hive 1 passing through congregation areas A, B, and C and showing evidence of convoluted flight at locations B and C. Sections of flight classified as straight are depicted in black; sections of flight classified as convoluted are shown by red lines. Gaps of greater than 30 s between consecutive data points are indicated by dashed lines. The start of the track is marked by a green triangle and the end by a red rectangle. Hives are marked by blue circles and numbered. The center of mass of each cluster of data points that we identified as a probable drone congregation area is marked by a gray circle and labeled A-D. Convex hull polygons containing all data points assigned to each cluster are outlined in gray. Insets for each panel: zoomed view showing details of convoluted flight at congregation areas. Scale shown by bar in bottom left corner of panel. (B) Example flight from hive 3 showing convergence in both the route taken and the destination with the flight in A. (C and D) Example flights from hive 2 visiting congregation areas A and B and showing convergence in route and destination with the flights shown in other panels. Note that only the outbound portion of the flight in D is shown; either this drone did not return to the hive or the return flight was not detected. (E) Example flight from hive 1 showing a visit to congregation area D. (F) Example flight from hive 3 showing visits to congregation areas D, A, and B, with convoluted flight at D and A. See also Figure S2.

Drones were detected across the entire trackable area of the site, with high traffic corridors extending southeast and terminating in hotspots of high drone activity (Figure 1). We found drone activity was very similar in both years (Figures 1C and 1E). Drones from different hives converged on similar routes (Figures 2, S1, and S2). These apparently shared flyways do not necessarily indicate interaction between drones but may point toward use of similar heuristic rules to move around the landscape.

### Identifying potential drone congregation areas

Previous studies either sampled drones at discrete locations or used radar to monitor drone flight in the aggregate but could not identify or track the flight paths of individual drones (Loper, Wolf and Taylor, 1987, 1992). Consequently, little is yet known about the flight paths taken by individual drones. Our data show that drone flights typically consisted of periods of straight, direct flight interspersed with periods of convoluted, looping flight (Figure 2). We developed a simple algorithm to classify flight into straight and convoluted sections (Figure 2; see STAR methods). We identified 425 sections of convoluted flight in 329 flights (50.8% of all substantial flight segments). Multiple convoluted sections occurred in 67 flights (20.4% of all flights containing convoluted sections). The mean duration of convoluted sections of flight was 134.0 s ± 17.3 (mean ± standard error [S.E.], throughout). Among flights that contained convoluted sections, convoluted flight accounted for 56.5% ± 2.0 of the total flight duration.

We used a clustering algorithm to reveal geographically clustered activity in convoluted flights. We identified four clusters of drone positions with data points contributed by at least 10 different tracks (Figure 1B; Table S1). Examination of individual drone tracks confirms the importance of these probable drone congregation areas, with numerous flights approaching these areas along relatively direct flight paths and abruptly changing to convoluted flight (Figure 2).

### Orientation flight and route development

**Figure 3:**
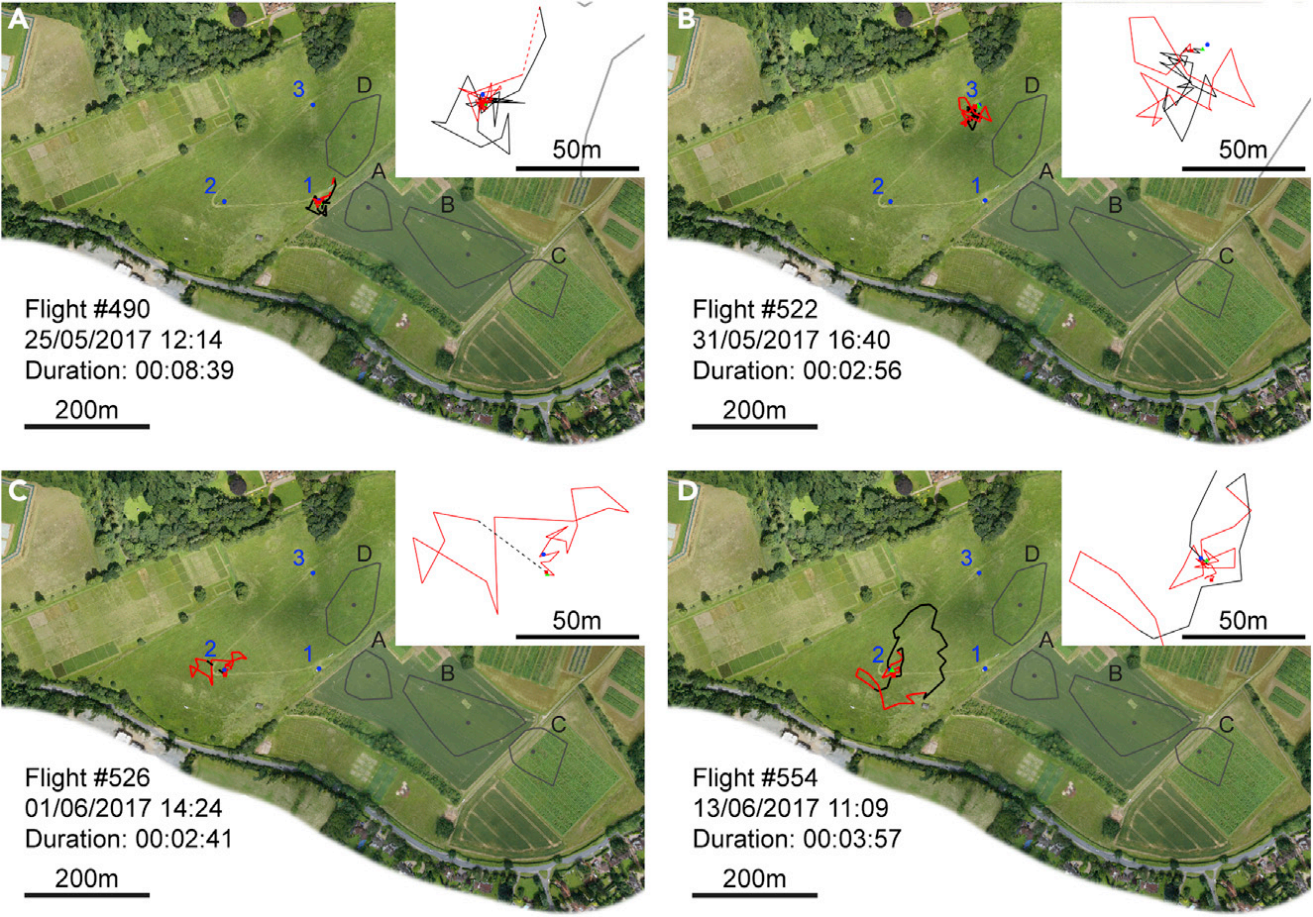
Orientation flights (A) Example flight path of the first flight (orientation flight) ever undertaken by a drone from hive 1. Sections of flight classified as straight are depicted in black; sections of flight classified as convoluted are shown by red lines. Gaps of greater than 30 s between consecutive data points are indicated by dashed lines. The start of the track is marked by a green triangle and the end by a red rectangle. Hives are marked by blue circles and numbered. The center of mass of each cluster of data points that we identified as a probable congregation area is marked by a gray circle and labeled A-D. Convex hull polygons containing all data points assigned to each cluster are outlined in gray. Insets for each panel: zoomed view showing details of flight path. Scale shown by bar in bottom left corner of panel. (B) Orientation flight of a drone from hive 3. (C and D) Orientation flights of two drones from hive 2, showing the typical range of distances reached from the hive. See also Figures S3 and S9.

We recorded 19 complete first flights of drones, comparable to orientation flights in workers (Capaldi et al., 2000). First flights remained close to the hive (mean maximum distance reached from starting position = 99.8 m ± 20.5) and frequently consisted of multiple loops in different directions from the hive (Figure 3). In this aspect, they more closely resemble the initial flights of bumblebee (*Bombus terrestris*) workers (Osborne et al., 2013; Woodgate et al., 2016), than honeybee workers, which typically perform a single loop per flight (Capaldi et al., 2000). Notably, drones performing orientation flights never undertook convoluted flight at congregation areas. The mean duration of first flights was 793.0 s ± 351.6.

**Figure 4:**
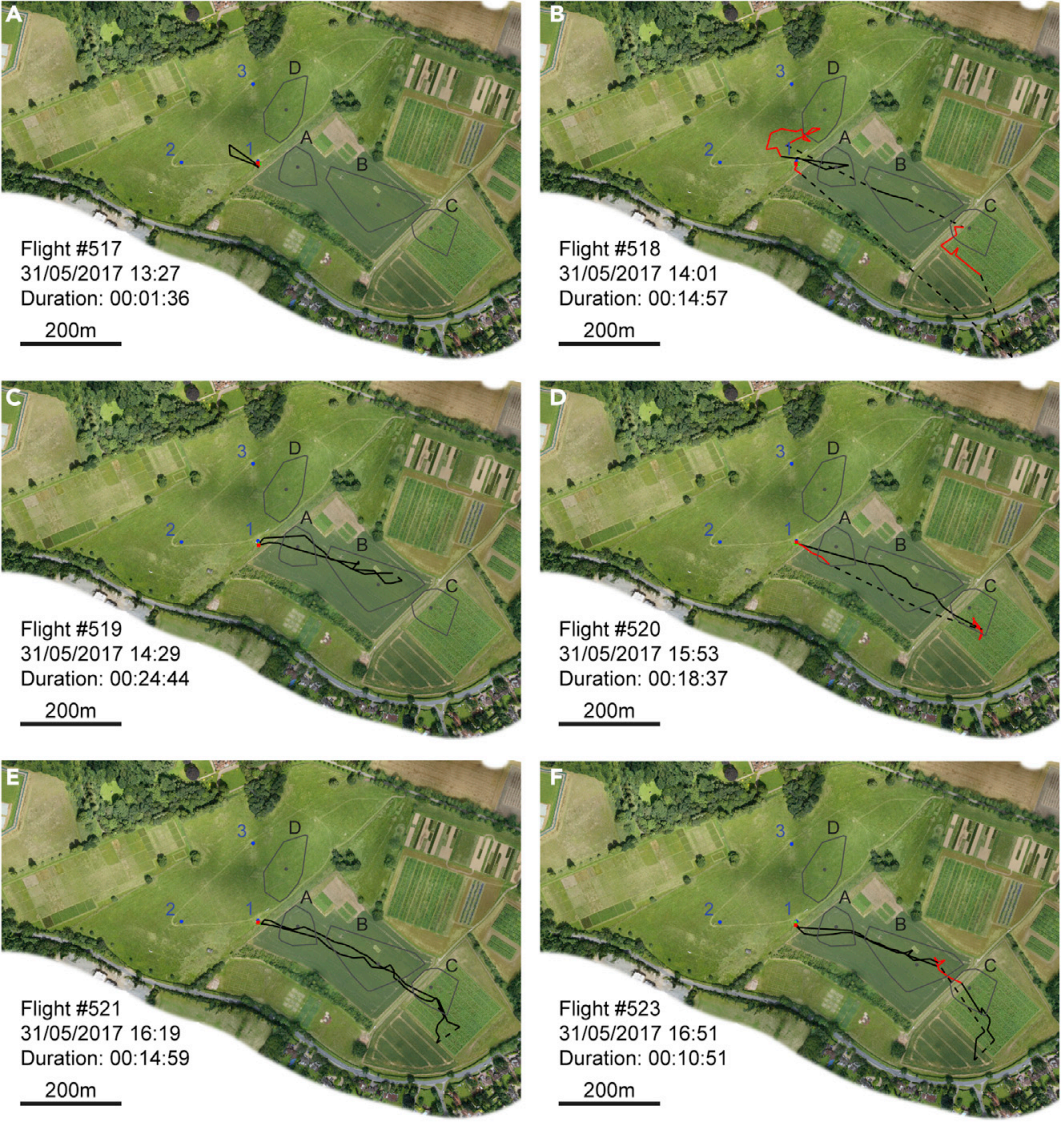
Example flight paths showing consecutive flights of drone #48 The first six flights ever undertaken by drone #48. Sections of flight classified as straight are depicted in black; sections of flight classified as convoluted are shown by red lines. Gaps of greater than 30 s between consecutive data points are indicated by dashed lines. The start of the track is marked by a green triangle and the end by a red rectangle. Hives are marked by blue circles and numbered. The center of mass of each cluster of data points that we identified as a probable congregation area is marked by a gray circle and labeled A-D. Convex hull polygons containing all data points assigned to each cluster are outlined in gray. (A) The drone's first ever flight was very brief: less than two minutes with convoluted flight directly in front of the hive entrance and a brief loop toward the Northwest. Scale shown by bar in bottom left corner of panel. (B) The second flight was much more extensive with loops passing through congregation areas D and A, followed by a longer flight through area C and appearing to continue even further, disappearing over a road that forms the southeastern border of our field site. The portions of flight we were able to detect were fairly straight, going directly to the congregation areas and showing no evidence of systematic search. (C–F) Subsequent flights by the same drone were even more direct, passing through congregation areas A, B and C, occasionally making convoluted flight at these locations, and apparently continuing across the road on two more occasions (E, F). See also Figures S3 and S9.

For four drones, we recorded 6–8 consecutive flights, beginning with their first ever orientation flight (Figures 4 and S3). Typically, one or two localized orientation flights were followed by an abrupt switch to flights traveling much further from the hive, passing through one or more congregation areas. Drones may thus need fewer orientation flights than typically undertaken by workers (mean 5.6 ± 2.9, Capaldi et al., 2000).

### Dynamics of drone flight at hives and congregation areas

Drones from all hives visited all four congregation areas in both years (Table S1), although area A was less commonly visited in 2017, while it is possible that the center of area C shifted southwards (Figures 1D and 1F). Among vertebrates with lek mating systems, males show high levels of fidelity to a single lek (Apollonio et al., 1989; Figenschou et al., 2004; Gibson et al., 2014; Fremgen et al., 2017). We found that it was common for drones to visit and perform convoluted flight at more than one congregation area during the same flight, connected by periods of much straighter flight (Figures 2A, 2D, and 2F). There were 154 flights which visited at least one congregation area (representing 23.8% of all flights recorded), 31 (20.1%) of which visited more than one congregation.

**Figure 5:**
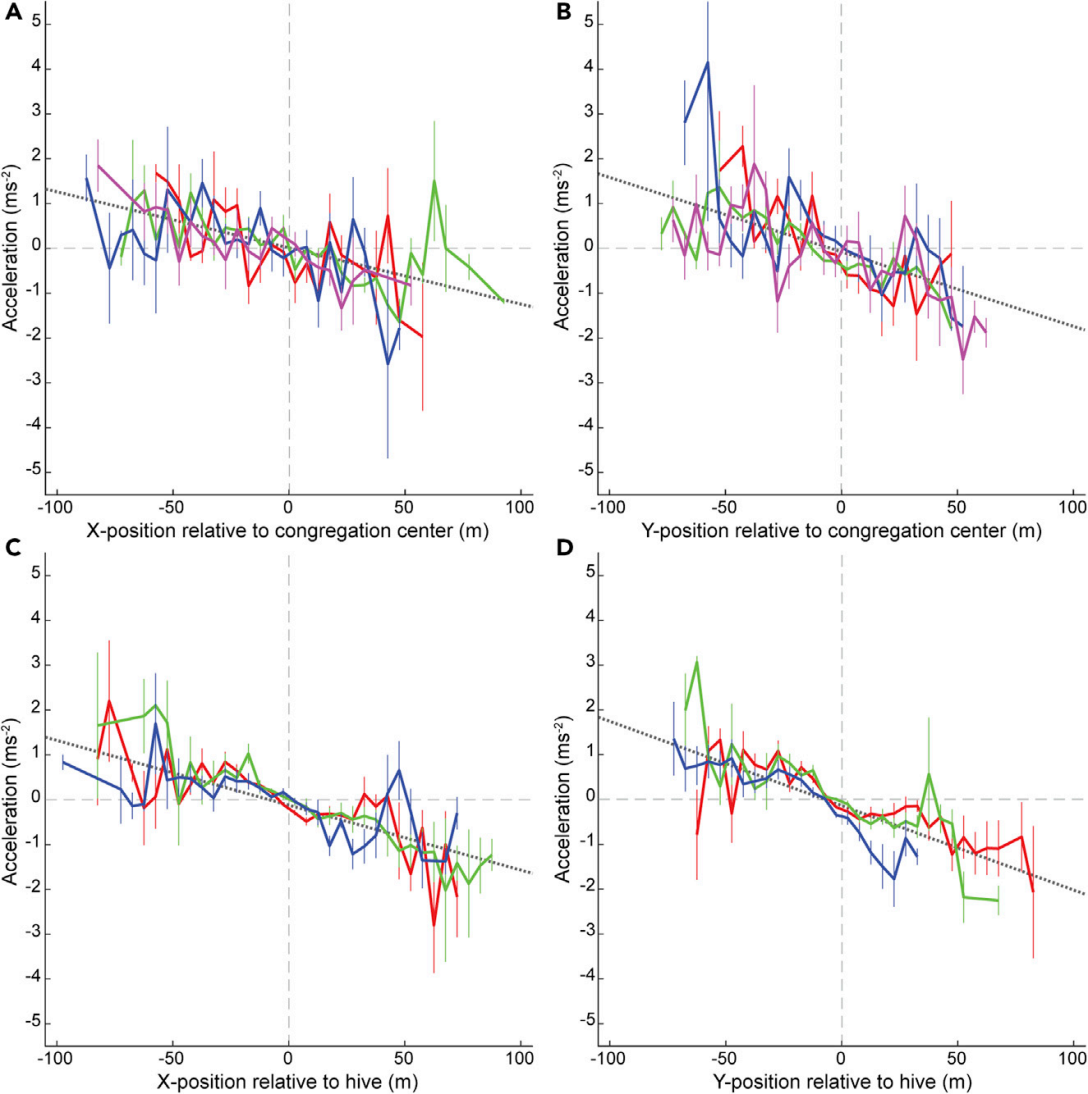
Mean acceleration as a function of position relative to the center of congregation areas or hives (A) Mean x component of acceleration calculated over bins of 5 m in the x direction (east-west) from the center of each congregation area. Red line: area A; green line: area B; blue line: area C; magenta line: area D. Narrow vertical bars show SE for each bin. Vertical dashed reference line indicates center of congregation area or hive. Horizontal dashed reference line indicates mean acceleration equal to zero. Gray dotted line shows regression line through all binned data. (B) Mean y component of acceleration (north-south) for the same locations. (C) Mean x component of acceleration calculated over bins of 5 m in the x direction from each hive location. Red line: hive 1; blue line: hive 2; green line: hive 3. (D) Mean y component of acceleration for the same locations. Scatter plots showing the full distributions at each location are shown in Figure S4. See also Figures S5 and S8; Tables S1 and S2.

We found a linear relationship between a drone's position relative to the congregation area or hive and its acceleration conditioned on its position, in both east-west and north-south directions of travel (all p < 0.005; Figures 5 and S4; Table S2). The x-intercepts (the location at which the acceleration is zero) were very close to the cluster center in all cases (mean ± S.E.: x direction = −1.36 m ± 2.52; y direction = −0.23 m ± 2.12; Table S2; Figure S4). In other words, the further drones moved from the center of a congregation area or hive during convoluted flight, the more strongly they accelerated back toward the center. Such patterns of acceleration function as an effective force—with individuals behaving as though they are trapped in an elastic potential well (Katz et al., 2011; Kelley and Ouellette, 2013)—and promote swarm cohesion (Kelley and Ouellette, 2013). Other characteristic properties of swarms, notably including midge mating swarms (Kelley and Ouellette, 2013), are that their distributions of velocity and position have Gaussian cores. This was true of our convoluted flight data at congregation areas (Figures S5 and S6). Taken together, these statistical properties of drone's convoluted flight suggest that this flight resembles swarming.

The relationship between position and acceleration appears linear within the region approximately ±20-50 m from each cluster center for both the x and y directions (Figure 5). Visual examination of the normal probability plots for x and y position (Figure S5) suggests that they deviate from Gaussian distributions at around ±30-50 m from the cluster centers. Taken together, these data suggest that drone congregation areas have roughly symmetrical cores of 30-50 m diameter.

**Figure 6:**
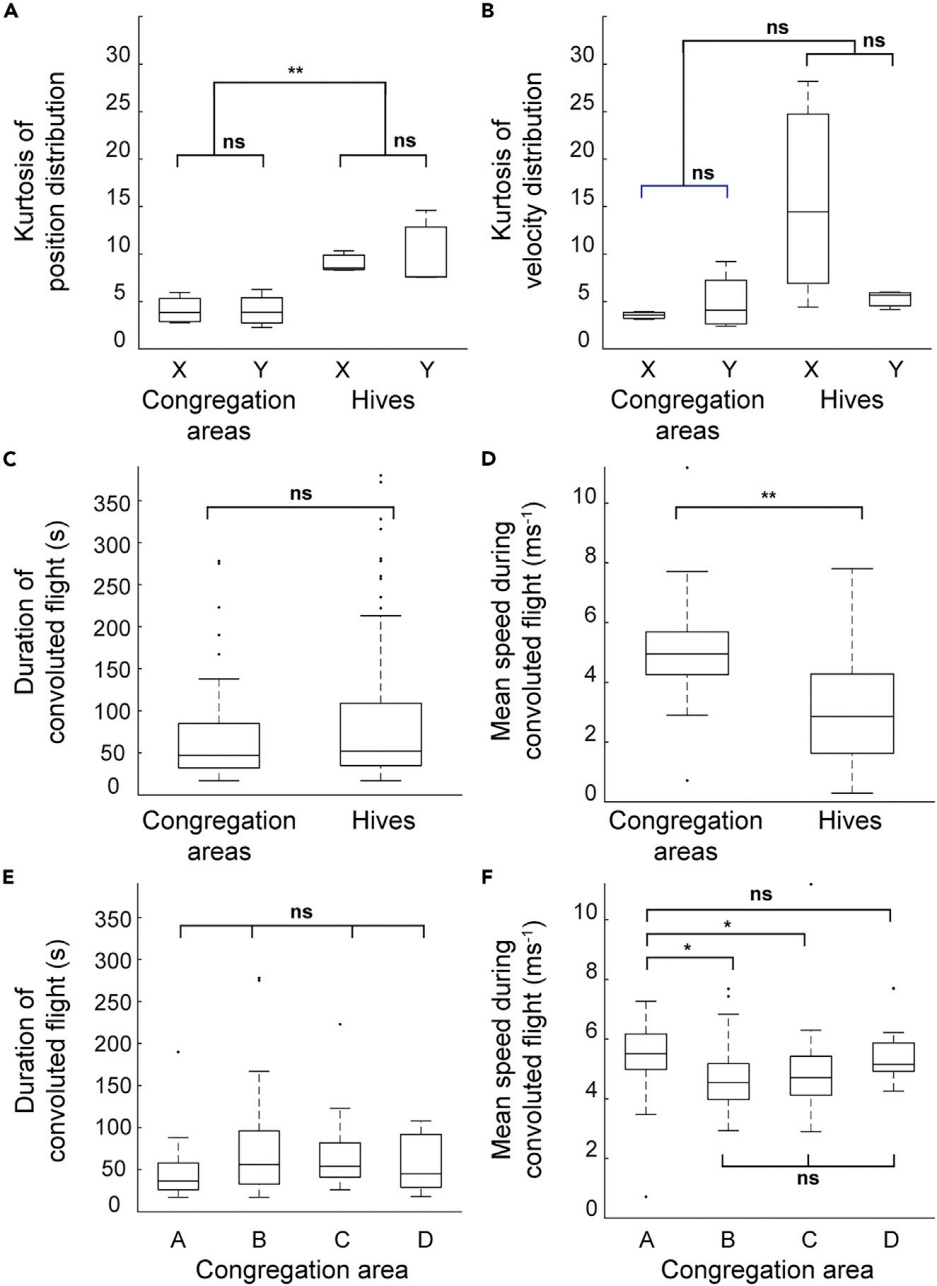
Differences in flight dynamics between convoluted flight sections occurring at congregation areas and those near hives (A) Box plots showing the kurtosis of distributions of drone positions in the x direction (east-west) or y direction (north-south) relative to the center of each congregation area or hive; flights at hives show significantly heavier-tailed distributions than those at congregations. Asterisks denote results of statistical analysis: ns: non-significant; ∗: p < 0.05; ∗∗: p < 0.01; ∗∗∗: p < 0.001. (B) Box plots showing kurtosis of distributions of drone velocity in the x and y directions. (C) Box plots showing the duration of convoluted sections of flight whose center of mass lies within 50 m of the center of a congregation area or of a hive. Only hive sites that were populated at the time the convoluted section occurred are included. (D) Box plots showing mean speed of flight during convoluted sections of flight at congregation areas or hives; flight in the congregations was significantly faster than that at hives. (E) Box plots showing duration of convoluted flight at each congregation area. (F) Box plots showing mean speed of sections of convoluted flight at each congregation area; bees flew faster at area A than at areas B or C. See also Table S1.

The dynamics of flight at congregation areas differed from those at hives: the distributions of position and velocity, which at congregations resembled those of midge mating swarms, have much smaller cores in the case of flight at hives (Figures S7 and S8). We tested for a difference in kurtosis, a measure of how “heavy-tailed” each distribution is. The kurtosis of the position distributions for flight near hives was significantly greater than that for congregation areas (F_1,6_ = 19.31, p = 0.007; Figure 6A), while the velocity distributions showed a similar but non-significant trend (F_1,6_ = 3.76, p = 0.110 Figure 6B). There was no effect of direction (x or y) on the kurtosis values (position: F_1,6_ = 0.10, p = 0.765, Figure 6A; velocity: F_1,6_ = 1.09, p = 0.338; Figure 6B).

Flight at congregation areas was significantly faster than at hives (congregation areas, 5.05 ms^−1^ ± 0.14; hives, 3.03 ms^−1^ ± 0.10; F_1,303_ = 15.73, p = 0.008; compare to mean speed of straight flight sections, 4.80 ms^−1^ ± 0.08, Figure 6C), but there was no difference in the duration of convoluted flight sections (congregation areas, 111.4 s ± 25.2; hives, 141.6 s ± 23.3; F_1,303_ = 0.45, p = 0.515; Figure 6D). These results demonstrate that the convoluted flights recorded at congregation areas differ in their flight dynamics from those around hives, likely reflecting different functions, with flight near hives probably not a form of male aggregation.

There were no significant differences between the four congregation areas in the duration of convoluted flight sections (F_3,80_ = 0.67, p = 0.574; Figure 6E; Table S1), but the mean speed of convoluted flight sections at congregation area A was greater than at areas B or C (F_3,80_ = 4.63, p = 0.005; pairwise comparisons using Tukey's method: A vs B, p = 0.016; A vs C, p = 0.035; all other pairwise comparisons, p > 0.05; Figure 6F).

### Virgin queen flight

We attempted to track the flight of virgin queens for comparison, across three years, but with little success (Figure S9). Two of three queens in 2016, 13 of 27 queens in 2017, and 11 of 64 queens in 2019 were recorded flying. First flights bore a strong resemblance to the first flights of drones: multiple local loops, centered on the hive, but remaining even closer to the hive than drones (mean maximum distance reached from starting position = 32.2 m ± 5.8; mean time in flight = 342.7 s ± 52.3; Figure S9C). Few queens undertook more than a handful of flights (maximum number of flights by a single queen = 6; mean number of flights per queen = 2.7 ± 0.3), but the subsequent flights we did record largely looked similar to first flights (N = 44; mean maximum distance from starting point = 43.5 m ± 12.9; mean time in flight = 270.1 s ± 39.7; Figure S9B). Only a small number of flights deviated from this pattern. Queen #04 on its third flight, on 20/06/2017, undertook a flight lasting 14:52, much of which was not detected by the radar, but in which it traveled at least 235 m from the hive and was detected at various angles from the hive, suggesting a broadly looping flight pattern (Figure S9D). Queen #10 on its second flight, on 25/08/2017, flew at least 133 m north of the hive location, toward the position of drone hive 3 (Figure S9E). Part of its flight was not picked up by the radar but the longest gap in the track is 2:02, so it is unlikely to have flown much further. This queen was observed to have mating sign on her return and, given the shortness of the gaps in the flight track, probably mated near to drone hive 3, without visiting any drone congregation area. Queen #20 on its first flight, on 16/07/2019, undertook a flight in which, after almost no hovering in front of the hive entrance, it flew rapidly east toward drone congregation area A but dislodged its transponder and could not be tracked further (Figure S9F). Queen #20 returned to the hive 30 min later and was observed to have mating sign. Queens #10 and #20 were the only queens in our experiment that could be confirmed to have mated and it seems unlikely that any other than the flight of queen #04, described above, could have had the opportunity to mate. Queens appear to be less amenable to radar tracking than drones or workers and until these challenges can be overcome, it is not possible to draw conclusions from these tracks regarding where queens search for mates.

## Discussion

Using harmonic radar tracking, we have recorded the behavior of individual honeybee drones as they explore the landscape and search for mates, revealing a characteristic switch between relatively straight periods of flight to a tightly looping pattern, often multiple times in the same flight. These individual tracks show the signature of collective behavior: convoluted flights were clustered in four areas of our experimental site, and the flight dynamics of drones suggest the mechanism by which group cohesion is maintained, demonstrating that these areas are swarms (Kelley and Ouellette, 2013). These results reveal the internal structure of drone congregation areas (Taylor, 1984; Koeniger et al., 2005a, 2005b; Galindo-Cardona et al., 2012).

It was common in our study for drones to visit more than one congregation area within a single flight: a fifth of flights in which drones undertook convoluted flight at a congregation area, or lingered in the area too long to be merely passing through, went on to visit other congregations. Travel between neighboring areas was particularly common, perhaps facilitated by their locations on shared flyways (Loper et al., 1992). Bouts of convoluted flight in our data set were relatively short, with a mean duration of little over two minutes, perhaps suggesting that drones routinely patrol between swarm locations, lingering only briefly in each to search for the presence of a queen.

The dominant hypothesis for the purpose of congregation areas is that they function akin to leks (Zmarlicki and Morse, 1963; Baudry et al., 1998; Koeniger et al., 2005a). Among lekking species of birds, mammals, and fish, individual males show a high degree of fidelity to a particular lek site (Apollonio et al., 1989; Figenschou et al., 2004; Gibson et al., 2014; Fremgen et al., 2017). Switching between leks is rare (Fremgen et al., 2017), and regular movement between leks within a day, or even a breeding season, is unknown. Males of many insect species form dense, lek-like aerial swarms, above visual cues known as swarm markers, near treetops, or at hilltops (Sullivan, 1981; Alcock, 1987; Shelly and Whittier, 1997; Van Veen, Sommeijer and Meeuwsen, 1997). These often maintain a relatively stable size and shape even as individuals leave and others arrive, leading Sullivan (1981) to hypothesize that individual males move between adjacent swarms. There is no previous experimental support for this hypothesis; however, one study suggested that male mosquitos were faithful to a particular swarm over a period of several days (Nielsen and Nielsen, 1953). Our radar tracks provide the best evidence for a mating strategy in which individuals travel between multiple aerial leks whose locations are fixed. Tracking or capture-mark-recapture studies of other swarming insects may reveal similar movements between swarms.

We identified four apparent congregation areas, each of which was visited by drones from all three hives and across both years of tracking. Nonetheless, there were some differences between them: areas B and C were frequently visited in both years but area A was much less visited in 2017 than in 2016, and flight speeds during convoluted flight at area A were higher than those at B or C. Although area D was visited as often as area C, a high proportion of visitors came from hive 3 and passed through en route to areas B and C. It is possible that while some congregation areas remain stable from year to year and are defined by the features of the landscape, others may be less permanent and influenced by the positions of colonies or other factors. Loper et al. (1992) reported occasional transient “bubbles” of drone activity within flyways, but areas A and D in our study appear to be more stable than that, with activity recorded in both areas over two years. Further work may reveal whether the term drone congregation area presently confuses multiple discrete phenomena.

Our results on flight dynamics explain how congregations can remain stable, even though individual drones do not remain there for prolonged periods: the relationship observed between acceleration and distance from the center will tend to function to draw individuals back in toward the center, creating an emergent potential well that keeps drones bound to the congregation (Okubo, 1986; Kelley and Ouellette, 2013). The congregation thus takes on physical properties, emerging from the collective behavior of the individuals within it. Drones thus use the same mechanisms for swarm cohesion as midges or mosquitos but on a far larger spatial scale (our congregations had a radius of approximately 50 m, compared to approximately 10 cm for swarms of *Chironomus riparius* midges [Kelley and Ouellette, 2013]). Individual drones tended to perform convoluted flight for 2–3 min at a time, but if drones leaving the congregation are replaced by newly arriving ones, the congregation itself can remain stable for far longer periods (Sullivan, 1981).

The congregation areas and flyways we have identified were frequented by drones across two years, demonstrating, in concert with the results of Loper et al. (1992), that swarms in relatively restricted volumes can remain stable over multiple years. This adds perspective to previous reports that the broad areas of drone activity revealed by lure sampling studies persist over long periods (Strang, 1970; Ruttner and Ruttner, 1972). No individual drones could possibly visit a drone congregation area in multiple years since they do not survive over winter. The locations of drone congregations, therefore, must be discoverable by individual drones rather than being learned from others. Our data show that orientation flights of drones typically do not take them far enough from their hive to discover congregations and that drones switch from orientation to making direct flights to congregation areas within one or two flights, without obvious signs of systematic searching. Cues to congregation area locations must be perceivable from relatively close to the hive, and since drones from all hive locations visited the same congregations, they must be perceivable from many locations. Previous authors have suggested several landscape properties that might determine where drone congregations form: low parts of the skyline (Ruttner and Ruttner, 1966, 1972), distance from tree cover (Zmarlicki and Morse, 1963; Ruttner and Ruttner, 1966; Galindo-Cardona et al., 2012), and south facing aspect (Galindo-Cardona et al., 2012). None of these, however, are sufficient to predict exactly where swarms will form. Our flight tracks demonstrate that drones share routes through the landscape, as well as destinations, and these flyways (Loper et al., 1992) remained stable over two consecutive years and so are likely also to be determined by the structure of the landscape. Flyways might play a role in helping drones locate congregations, potentially explaining why it has proved so difficult to find any combination of cues that defines individual congregation areas. Reconstruction, from radar track data, of the views experienced by drones as they navigate to and from drone congregation areas promises to reveal the cues they use.

It has been long hypothesized that drones gather in large numbers at drone congregation areas (Taylor, 1984; Koeniger et al., 2005a, 2005b; Galindo-Cardona et al., 2012), but this has been challenged (Butler and Fairey, 1964; Currie, 1987) because almost all evidence for these congregations comes from studies using either caged queens or pheromone lures to attract drones. Such studies cannot with certainty refute the alternative hypothesis that these sampling methods, themselves, cause the congregations. This debate was partially resolved when Loper et al. (1992) used radar tracking to demonstrate that drones congregated in repeatable locations in the absence of lures. However, their observations departed from the consensus emerging from lure-sampling studies in several ways: the clusters of activity that Loper et al. identified as drone congregation areas were much smaller than previously assumed (100 m diameter, with a peak of 68 drones observed at any one time [Loper et al., 1992]; compared to 220 m × 260 m during the South African winter, enlarging to 500 m × 1000 m in summer [Tribe, 1982]; a mean of 11,750 drones estimated at a single congregation using lure sampling [Koeniger et al., 2005a]) and were found much closer together. Loper et al. (1992) also suggested that shared flyways around the landscape might be more important than the congregations themselves. They were unable to track individuals, but our work now corroborates most of their unusual findings: using different methodology, we also estimated our congregations to be approximately 100 m across and identified shared flyways between them. We found four such locations at close proximity. The placement of congregations B and C, either side of a roadway, appears to agree with the suggestion that congregations form where terrain features are interrupted (Loper et al., 1992).

Why do radar studies of drone activity depart from the observations of lure sampling studies? The most likely explanation is that the superior spatial and temporal resolution of radar monitoring has revealed the internal structure present in drone congregation areas. We suggest that the locations described as drone congregation areas by previous authors (Zmarlicki and Morse, 1963; Taylor, 1984; Koeniger et al., 2005a, 2005b; Galindo-Cardona et al., 2012) are likely to actually comprise several distinct swarms and their associated flyways. Our data demonstrate that these substructures and not just the broad region favored by drones are themselves stable over a timescale of years. If, as our data suggest, individual drones move between congregation areas, remaining for only short periods at each, the congregations may never have more than a small number of drones present at once. Aerial traps, though, will catch not only drones present when the lure is raised but all those that subsequently arrive (while few are able to leave), gradually depleting the population of an entire network of congregations and flyways. This may also partly explain why the supposedly enormous aggregations of drones have proven difficult to locate when much smaller swarms of midges, mosquitos, or wasps are readily discovered (Sullivan, 1981; Shelly and Whittier, 1997). Another explanation for the discrepancies between radar and lure sampling studies could be that the presence of queens or pheromone lures alters drone behavior sufficiently to interrupt the normal structure of congregation areas, causing them to expand or perhaps inducing several, ordinarily distinct congregations to merge (Ni and Ouellette, 2016). Careful experiments using radar to monitor drone activity in the presence of lures could resolve the question of whether congregations are smaller in the absence of lures or whether drone congregation areas have an internal structure which radar tracking is only now starting to reveal.

### Limitations of the study

Due to the logistical problems involved in moving the harmonic radar, we monitored the movements of drones in just one location. We partially mitigated this issue by tracking bees from three different hives, demonstrating that the behaviors we uncovered are not completely idiosyncratic to a single spatial location, but the three hives were close enough that bees from each encountered a substantially similar landscape. Repetition of this work in other locations will establish how the networks of flyways and stable congregation areas identified in our work and by Loper et al. (1992) are influenced by landscape structure. Loper er al. (1992) found that flight at congregation areas took place at higher elevations than that in flyways, although drones were rarer and rarer as elevation increased. We angled the harmonic radar to maximize our ability to track bees across the entire network of flyways and congregations, so it is likely that further flight activity took place at congregation areas too high for us to detect. Current harmonic radar technology does not allow us to identify individual bees when several transponders are used. Solving this problem would open up the potential to investigate interactions between drones and between drones and queens.

## STAR★Methods

### Key resources table

**Table:** 

REAGENT or RESOURCE	SOURCE	IDENTIFIER
**Deposited data**		

Deposited data	This study	https://doi.org/10.6084/m9.figshare.14462073

**Experimental models: Organisms/strains**		

European honeybee drones, *Apis mellifera*	Colonies managed by Rothamsted Research, UK	N/A
European honeybee queens, *Apis mellifera*	Honeybee breeders, Hertfordshire, UK	N/A

**Software and algorithms**		

Matlab code used for data processing and analysis	This study	https://doi.org/10.6084/m9.figshare.14462070
Matlab, version R2018a	Mathworks inc., Natick, USA	www.mathworks.com

### Resource availability

#### Lead contact

Further information and requests for resources should be directed to the lead contact, Joseph Woodgate (j.woodgate@qmul.ac.uk).

#### Materials availability

This study did not generate new unique reagents or other new materials.

#### Data and code availability

Original data and code generated during this study have been deposited to Figshare (figshare.com): data, https://doi.org/10.6084/m9.figshare.14462073; code, https://doi.org/10.6084/m9.figshare.14462070.; see key resources table.

### Experimental model and subject details

#### Apis mellifera

We tracked adult drones (males) of the European honeybee (*Apis mellifera*), from 8 days after eclosion until they died or did not return to their hive (mean age at death: 21 days, [Witherell, 1971; Reyes et al., 2019]). All drones were produced by honeybee colonies at Rothamsted Research, Harpenden, Hertfordshire, UK. We attempted to track 297 drones from 2016-2017. We also attempted to track 94 adult, virgin *A. mellifera* queens from 2016-2019, from approximately one to six weeks after eclosion. All queens were obtained from local queen breeders in Hertfordshire, UK. Experiments were conducted with approval from the Rothamsted field experiments committee.

#### Housing of drone colonies

In both 2016 and 2017, we used three colonies of honeybees, each housed inside a shed, from which they could freely access the outside world through a Perspex tunnel. Different colonies were used in each year. Throughout this manuscript *hive 1*, etc., is used to describe the location of each hive, not the identity of the bee colony. The lowest level of the hive housed a custom-made observation frame with Perspex walls through which we could observe the bees. Crucially the Perspex walls were approximately 20 mm from the comb, rather than the usual 9 mm “bee space”, allowing a drone with a radar transponder on its thorax to move freely. A queen excluder (4.3 mm mesh, through which workers can pass but the larger queens and drones cannot) was placed above the observation frame to prevent drones with attached transponders accessing the rest of the hive, where they would get trapped in the smaller spaces.

#### Housing of queens

We tracked three queens over 9 days in August 2016, and a further five over 20 days in June 2017. These queens were obtained from a commercial queen-breeder. Shortly after emergence, we permanently glued a radar transponder to the thorax of each queen, as for drones (see STAR methods). Each queen was introduced to a 2-frame observation hive along with 50-100 workers and sited in one of the three sheds next to the drone hives, with their own clearly marked entrance hole. Observation hives had approximately 20 mm of space between the comb and outer wall, to allow the queens to move with transponders attached (see STAR methods). Entrance tunnels were permanently open so that queens and workers could come and go at will. Only one queen was present at each hive location, so tracks could be uniquely identified.

Observations of queens in observation hives suggested that the retinue of workers surrounding the queens made it difficult for them to move with transponders attached, so over 45 days from August to September 2017 and 63 days from June to August 2019, we tracked a further 86 queens which did not have transponders permanently attached. Instead, a thin metal disc (4mg, Ø2mm, Qualitech, March, Cambridgeshire, UK) was permanently glued to the thorax of each queen. Each queen was introduced to a small mating nucleus box (Apidea Vertriebs AG, Cham, Switzerland) with approximately 100 workers. These boxes were placed side by side on a table beneath an open-sided gazebo (3 m x 3 m by 4 m high, Gala Tent Ltd, Rotherham, UK), to provide protection from rain (location: 2017, 51° 48.2145’N, 0° 22.1458’W; 2019, 51° 48.1974’N, 0° 22.1877’W). Each mating box was fitted with a small Perspex tunnel (30mm wide x 30mm high x 60mm long) containing a queen excluder: workers could thus come and go freely but queens could not leave the box.

#### Drone breeding and identification

We encouraged the production of drones by placing drone comb in the upper stories of the hive and placed a queen excluder between the brood frames and the lower story of the colony, ensuring that the drones were unable to access the entrance and leave the hive. The colonies were checked daily and any newly emerged drones marked with colored paint pens (Posca PC-5M, Mitsubishi Pencil Co., Japan), allowing us to track their ages. Once drones were older than 8 days, the mean age at first flight (Ruttner and Ruttner, 1966; Witherell, 1971), they were moved in small groups to the observation frame and had a radar transponder attached permanently with superglue (Loctite Power Flex Gel, Henkel Ltd., Hemel Hempstead, UK). Transponders consist of a 16 mm vertical dipole and weigh around 15 mg.

When previously inexperienced drones were unavailable, we instead tracked drones obtained from two other sources: drones from our observation hives whose age and previous flight experienced were unknown, or drones captured at another apiary, approximately 6.25 km from the experimental site. These drones were caught at the entrance of the offsite apiary when returning from flights so definitely had previous flight experience, although their age and the extent of their experience was unknown. They were transferred to one of the experimental hives (allocated at random) in a darkened container and tracked from their first experience at the experimental site.

### Method details

#### Field site

The study was carried out in a field of grass in an agricultural landscape at Rothamsted Research, Harpenden, UK, AL2 2JQ.

#### Harmonic radar

Drones with transponders attached were free to come and go from the hive, and we used 32 mm harmonic radar (Riley et al., 1996; Woodgate et al., 2016, Woodgate et al., 2017) to monitor any flights that took place. The radar returned distance and azimuthal direction coordinates of the position of any detected transponder, every 3 s while the bees remained in line-of-sight within a radius of about 800 m. These polar coordinates were converted to GPS coordinates by triangulation, using two locations of known GPS position (accuracy approximately ±2 m; in theory azimuthal accuracy will decrease with distance, but we have not observed a decline in accuracy over the range of distances in our data).

We attached transponders to 104 drones in 2016 and 193 drones in 2017. The radar system cannot identify individual transponders, so it was not always possible to assign a unique identity to every radar track, particularly if multiple individuals were active in the same area at the same time. Many drones whose tracks were recorded could be identified from video footage of the hive entrances (using Sony Handicam HDR-CX240, Sony Corporation, Tokyo, Japan); from the observations of someone stationed at the entrance to the hive; or by deduction when only one drone was present in a hive, or the locations of all but one drone were known. Flights could be assigned to 78 unique individuals across both years. Many other tracks could not be assigned to a particular individual.

All the data points that could be confidently assigned to a single flight by a single individual (which can often be done, even if we did not know the identity of that individual), were considered to make up a *flight segment*. Flight segments do not necessarily constitute a complete record of a complete flight by a drone, since a bee’s position often cannot be determined if it lands on the ground or flies too high or low, leaves the trackable range of the radar, or if it enters the radar shadow cast by large objects such as trees or buildings. Additionally, there were a few occasions when multiple bees were active in the same area at the same time, and it was not possible to determine which one was the source of each signal.

We recorded 1174 flight segments in total over two field seasons. Any flight segment which lasted less than 30 s or in which the bee moved less than 15m from its starting position was considered to be too short to reveal the characteristics of drone flight; these segments were included in the dataset used to make heat map figures, since they still provide evidence regarding which parts of the landscape were frequented, but they were excluded from further analyses. This left 648 *substantial flight segments*. Of these, 225 substantial flight segments represented complete flights, starting and ending at a hive. A further 156 recorded the outbound portion of flights starting at a hive, but did not show the return flight, while 116 ended at a hive but were missing the outbound track; the remaining 151 flight segments could not be unambiguously assigned to a hive.

#### Tracking queens

All queen mating boxes were monitored constantly from approximately 9:30 to 18:00. When a queen entered the tunnel of her box, a radar transponder with a small magnet glued to the base (neodymium, 12 mg, Ø2 mm, Magnet Expert Ltd, Tuxford, UK) was quickly attached to the metal disc on the queen’s thorax. The tunnel was then opened and the queen’s flight monitored using harmonic radar. Only one queen was allowed to depart at a time; any others attempting to leave during this time were blocked by the queen excluder. All queens were monitored on their return for the presence of mating sign, an indicator of successful copulation (Koeniger, 1990).

### Quantification and statistical analysis

#### Heat maps

We visualized our recorded drone flights as heat maps (see Figures 1, S1, and S10). First, we selected a dataset to map (e.g. all recorded drone flights; or all flights recorded in 2016). The field site was broken down into 5 m x 5 m pixels. For each pair of consecutive radar locations recorded from the same bee, we used a method derived from Brownian Bridge Movement Models (described in detail in Woodgate et al., 2017), to estimate the probability that the bee passed through each landscape pixel during the transition from one fix to the next. Transitional periods were broken down into 5 *timeslices* per second, and the probability density function calculated for the bee’s position at each timeslice. This procedure results in an estimate of the likelihood of the bee having passed through each pixel in the time between the two radar fixes, for which we know its position with certainty. The probability maps thus generated for each timeslice were summed across all timeslices in a flight segment to calculate the probability that the bee passed through any given pixel at any point during the flight.

For each flight segment, we normalized these probabilities such that the ‘hottest’ pixel in each segment had the same value. This prevents a small number of tracks from disproportionately influencing the overall heat map: multiple tracks must visit the same pixel to produce a hotspot. For each pixel, we then summed the normalized probabilities obtained over the entire dataset, obtaining a count-like estimate of how often that pixel is likely to have been flown over, compared to other pixels. Each pixel was colored proportionally to its sum total, scaled to fit an aerial orthomosaic image of our field site and plotted on top of the orthomosaic image. Note that the colors in each heat map image are scaled to fit the data: within a single image, ‘hotter’ white and yellow regions are more visited than ‘colder’ red and black ones, but they cannot be used to compare the number visits to each region between datasets. Transparency was also added to the pixel image to allow the field site image to be seen beneath it: any pixel whose sum total was less than the 1^st^ percentile of the entire distribution is entirely transparent; any pixel whose sum total exceeds the 5^th^ percentile is entirely opaque; and the transparency of pixels lying between those limits is proportional to their sum totals.

#### Identifying convoluted flight

We divided each recorded flight segment into *sections* of flight that were characterized as either *straight* or *convoluted*. First, the difference in coordinates of every consecutive pair of data points was used to determine a bearing for every transition between positional fixes (i.e. the angle between each pair of coordinates). We then calculated the resultant vector length of all the bearings falling within a moving 21 s window (7 rotations of the radar). Any data point that did not fall within a window whose resultant vector length exceeded 0.7 was a candidate for being part of a section of convoluted flight. This algorithm is scale free and makes no assumptions regarding the structure of convoluted flight other than that the bee does not consistently fly in the same direction. However, our dataset has frequent gaps where a bee was not detected on one or several sweeps of the radar and these can cause periods of flight that are not geographically or temporally adjacent to become combined in a single section of convoluted flight, so we imposed the following addition restrictions: no transition between data points in which there was a gap longer than 12 s and in which the bee’s position moved by more than 40 m could be included in a convoluted section. Finally, convoluted sections had to contain a minimum of 7 temporally consecutive data points. Straight sections were defined as all consecutive data points not included in a convoluted section. This algorithm has proven to be robust to variation in the exact values of the window-duration and vector length parameters (Figure S10). Example flight paths showing straight and convoluted flight sections are shown in Figures 2, 3, 4, S2, S3, and S9 and the results of this analysis were used to select the convoluted flight data whose analyses are presented in the Results section and in Figures 1, 5, 6, and S4–S8.

#### Identifying potential drone congregation areas

Having identified every positional fix in the dataset that could be attributed to a section of convoluted flight, we excluded all those belonging to a section whose *center of mass* (mean coordinates of every data point in the section) was within 50 m of any hive (since Zmarlicki and Morse (1963) reported that no drones could be attracted to a tethered queen 30 m from an apiary, but that they were attracted 60 m away). We used a clustering algorithm (the Matlab function *clusterdata*, using Euclidean distance with a cutoff of 8 m; Mathworks Inc., Natick, USA) to identify geographic clusters of data points. Because the clustering algorithm must assign each data point to a cluster, it returned 211 different clusters, but most consisted of only a small number of positional fixes, often drawn from a single section of flight. Since there is no reason to believe that every section of convoluted flight must occur at a drone congregation area, we applied the fairly conservative rule that a cluster must include data points from at least 10 different flight segments. This left us with four clusters as the candidate drone congregation areas. We defined the center of each congregation area as the center of mass of each cluster (mean coordinates of every data point assigned to the cluster). To indicate the approximate boundaries of these areas, we constructed convex hull polygons encompassing every data point assigned to each cluster. These are shown in Figures 1, 2 and 3, and S2, S3 and S9 for illustrative purposes only but were not used in the analysis of flight dynamics. The exact shape of a convex hull polygon depends on which data points are included in the dataset, but the centers of mass are far less affected by the inclusion or exclusion of a few data points on the perimeter and so are a more robust way to estimate congregation area locations.

#### Number of congregation areas visited

We identified tracks in which either the bee performed a section of convoluted flight whose center of mass (mean coordinates of every data point) was within 50 m of a cluster center, or in which the bee stayed within 50 m of a cluster center for at least 21 s (seven revolutions of the radar). This included periods in which the signal was lost, provided the positional fixes either side of the missing period were within 50 m of a cluster center: this can occur if the bee flies too high for the radar to detect. The number of different areas visited on each flight could be counted. The results of these analyses are presented in the Results section, with illustrative flight paths shown in Figure 2.

#### Flight dynamics

We assessed the flight dynamics of drones in each congregation area, as well as behavior around the hives. A section of convoluted flight was considered to take place at a congregation area or hive if its center of mass was within 50m of the center of the congregation, or the position of any hive that was in use at the time of the flight.

All positional fixes within a section were expressed relative to the center of the congregation area or hive. Movement in the x- and y-directions (East-West and North-South, respectively) was calculated as the difference, in meters, between consecutive x- or y-coordinates. Time differences were calculated as the difference, in seconds, between timestamps of consecutive data points. Components of velocity in the x and y directions were calculated as x- and y-movement divided by the time difference. Components of acceleration in the x and y direction were calculated as the difference between consecutive x- and y-velocities divided by the time difference.

We performed linear regressions on all data points of all sections whose center of mass was within 50 m of each candidate congregation area or hive (using Matlab’s *fitlm* function), with x- or y-position relative to the center of the congregation area or hive as the dependent variable and the x- or y-component of acceleration as the response variable. To visualize these data, we calculated the mean acceleration of all data points in 5 m bins from the center of each congregation area or hive. The results of these analyses are presented in the Results section and Table S2 and illustrated by Figures 5 and S4.

We investigated whether the kurtosis of position and velocity distributions differed between congregation areas and hives using two mixed-model ANOVAs (using the *anovan* function of Matlab), with the kurtosis values for position and velocity, respectively as the dependent variables. We tested for main effects of two categorical predictors (whether each distribution was for flights at a hive or potential congregation area, a between-subjects factor; and whether each distribution referred to position/velocity in the x- or y-dimension, a within-subject factor), with a code identifying each location (four potential congregation areas plus three hives) included as a random factor. The results of these analyses are presented in the Results section and illustrated by Figure 6.

We tested whether there was a difference in the duration or mean speed of convoluted sections at congregation areas or hives using two ANOVAs (using the *anovan* function of Matlab), in which the dependent variables were the total duration of each section and the mean speed of the drone during each section, respectively. We tested for the main effect of location category (whether each section of flight took place at a congregation area or hive), and included two random factors: the bee identity, and a code identifying each location (four potential congregation areas plus three hives). The results of these analyses are presented in the Results section and illustrated by Figure 6.

We tested whether there were differences in convoluted flight behavior between congregation areas using two ANOVAs (using the *anovan* function of Matlab), in which the dependent variables were the total duration of each section and the mean speed of the drone during each section, respectively. We tested for the main effect of congregation area identity and included one random factor: bee identity. We used Tukey’s method for pairwise comparisons (using Matlab’s *multcompare* function).
