# Supplementary material for: Harmonic radar tracking reveals that honeybee drones navigate between multiple aerial leks
Source: iScience. 2021 May 20;24(6):102499. doi: 10.1016/j.isci.2021.102499 (PMC8257961; doi:10.1016/j.isci.2021.102499)
Supplement: Document S1. Figures S1–S10 and Tables S1 and S2 [file mmc1.pdf]

iScience, Volume *24*

Supplemental information

Harmonic radar tracking reveals that honeybee drones navigate between multiple aerial leks

Joseph L. Woodgate, James C. Makinson, Natacha Rossi, Ka S. Lim, Andrew M. Reynolds, Christopher J. Rawlings, and Lars Chittka

Supplemental figures:

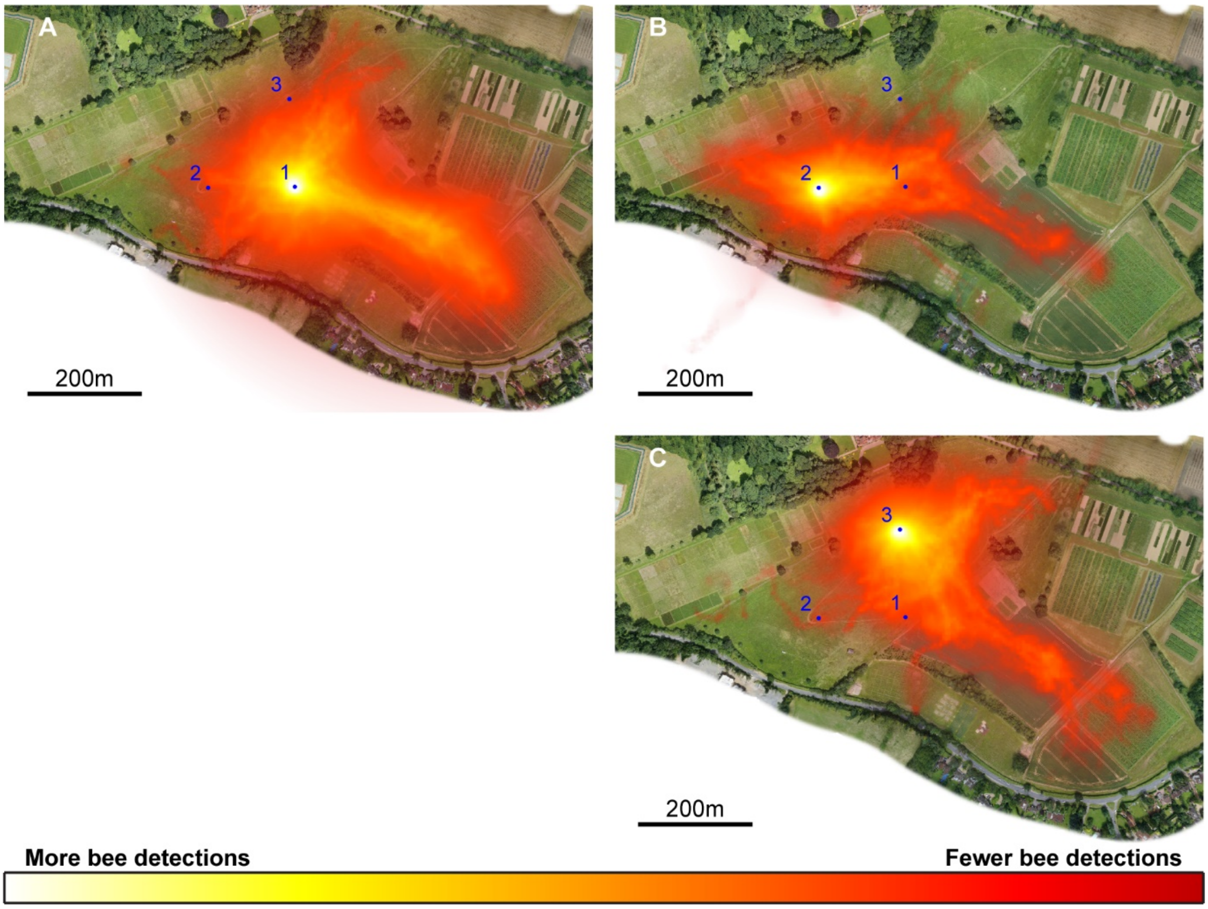

Figure S1. Heat maps showing drone activity broken down by hive of origin, related to Figure 1

A) Heat map showing all drone flights from hive 1, recorded over both years 2016-2017, superimposed on an aerial orthomosaic image of the field site. Hive locations are marked by blue circles and numbered. Areas with brighter, yellower colouration were more visited by drones. N = 256 tracks.

B) Heat map showing all drone flights from hive 2. N = 375 tracks.

C) Heat map showing all drone flights from hive 3. N = 131 tracks. High traffic corridors extended southeast, terminating in hotspots in the same locations. Flights from hives 2 and 3 show a distinct change of direction midway along the route taken to the hotpots in the southeast, allowing them to converge with the route used by drones from hive 1, even though both hives have a clear and unobstructed straight-line path available, implying the use of shared flyways (see also Figures 2, S2).

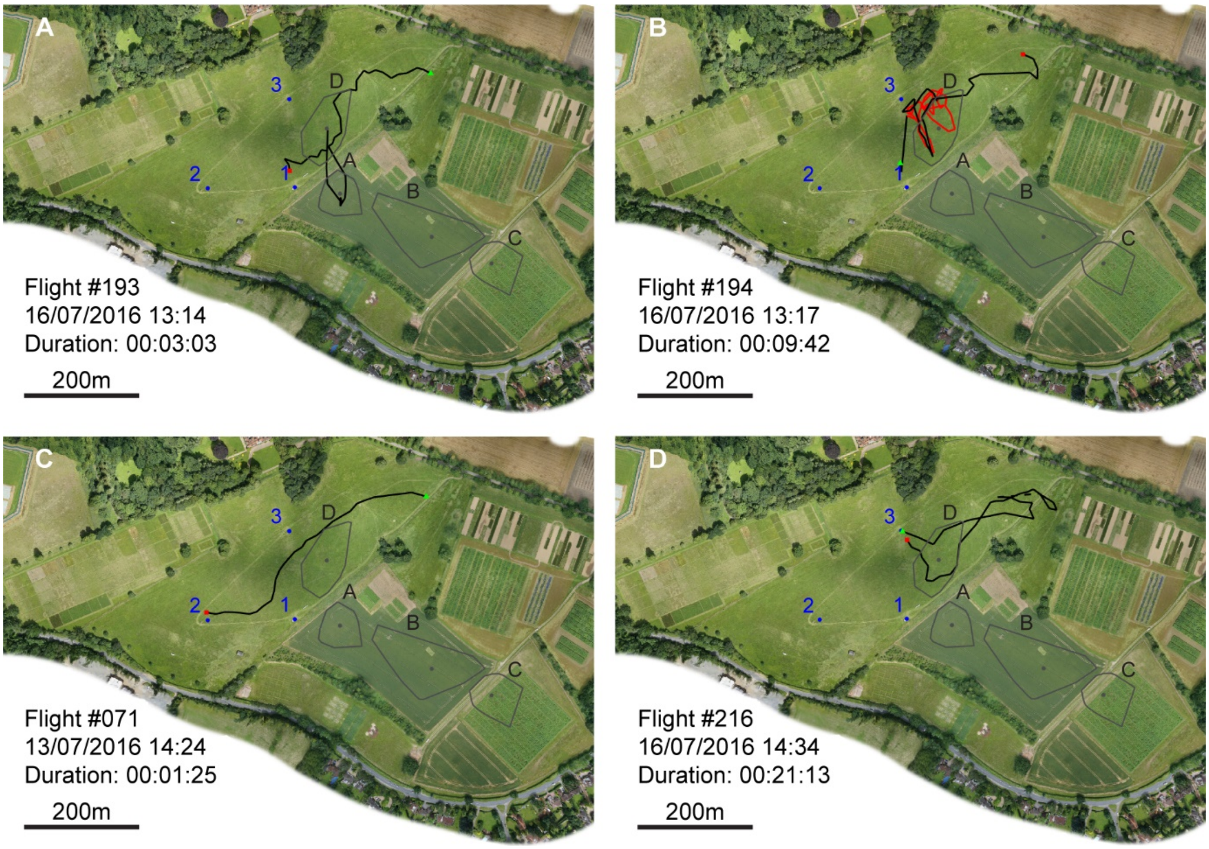

Figure S2. Example flight paths showing probable shared flyway, related to Figures 1, 2

A) Flight path of a drone from hive 1 returning from an unknown location to the northeast of the trackable area of the site. The outbound portion of this flight was not detected by the radar. Sections of flight classified as straight are depicted in black; sections of flight classified as convoluted are shown by red lines. Gaps of greater than 30 s between consecutive data points are indicated by dashed lines. The start of the track is marked by a green triangle and the end by a red rectangle. Hives are marked by blue circles and numbered. The centre of mass of each cluster of data points that we identified as a probable congregation area is marked by a grey circle and labelled A-D. Convex hull polygons containing all data points assigned to each cluster are outlined in grey.

B) Outbound flight path of a drone from hive 1, showing convoluted flight at congregation area D and leaving the trackable area to the northeast. Curved flight path shows convergence with track shown in A and is likely to be the same drone.

C) Inbound flight to hive 2 to destination to the northeast showing convergence in route and destination with flights from other hives in other panels.

D) Complete flight from hive 3 to destination to the northeast showing convergence in route and destination with flights from other hives shown in other panels.

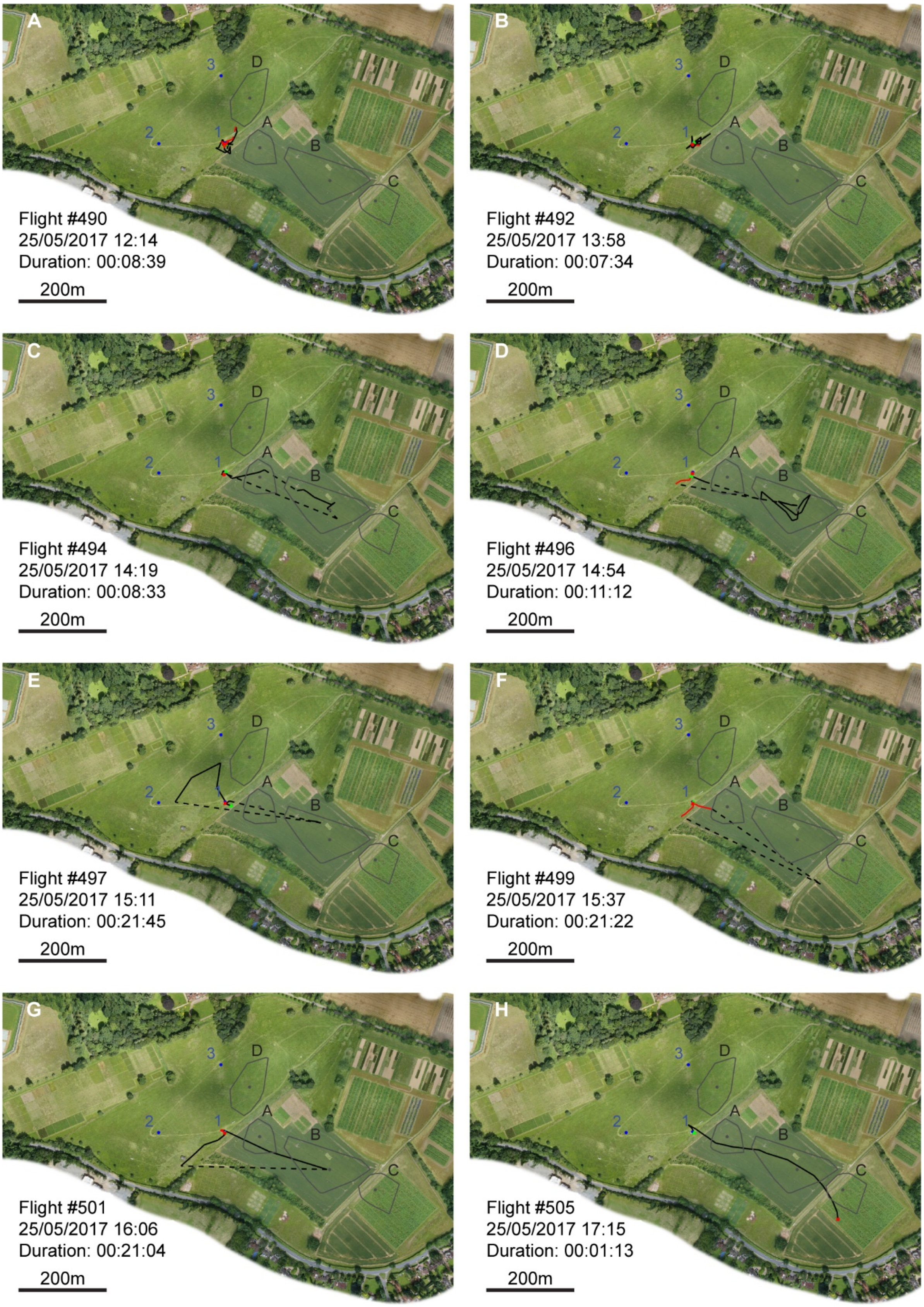

A
Flight #490
25/05/2017 12:14
Duration: 00:08:39
200m
B
Flight #492
25/05/2017 13:58
Duration: 00:07:34
200m
C
Flight #494
25/05/2017 14:19
Duration: 00:08:33
200m
D
Flight #496
25/05/2017 14:54
Duration: 00:11:12
200m
E
Flight #497
25/05/2017 15:11
Duration: 00:21:45
200m
F
Flight #499
25/05/2017 15:37
Duration: 00:21:22
200m
G
Flight #501
25/05/2017 16:06
Duration: 00:21:04
200m
H
Flight #505
25/05/2017 17:15
Duration: 00:01:13
200m

Figure S3. Example flight paths showing consecutive flights of drone #39, related to Figures 3, 4

The first eight flights ever undertaken by drone #39. Sections of flight classified as straight are depicted in black; sections of flight classified as convoluted are shown by red lines. Gaps of greater than 30 s between consecutive data points are indicated by dashed lines. The start of the track is marked by a green triangle and the end by a red rectangle. Hives are marked by blue circles and numbered. The centre of mass of each cluster of data points that we identified as a probable congregation area is marked by a grey circle and labelled A-D. Convex hull polygons containing all data points assigned to each cluster are outlined in grey.

A) The drone's first flight was typical of orientation flights, remaining close to the hive with convoluted flight centred on the hive location and no evidence of exploratory flight further afield.

B) The second flight was very similar in structure.

C) The third flight, taking place approximately 15 minutes after the drone had returned from its second flight, showed an abrupt change in structure. The track is missing some data, suggesting the drone flew too high or low for the radar to detect, but the data we do have show that the drone went much further from the hive, passing through congregation areas A and B. The portions of flight we recorded were fast and direct, with no evidence of orientation-flight-like convolution or of systematic search.

D-G) The remaining flights by this drone were very similar: direct flights passing through congregation areas A and B.

H) The drone did not return from its eighth flight; it is unknown whether it mated successfully or died.

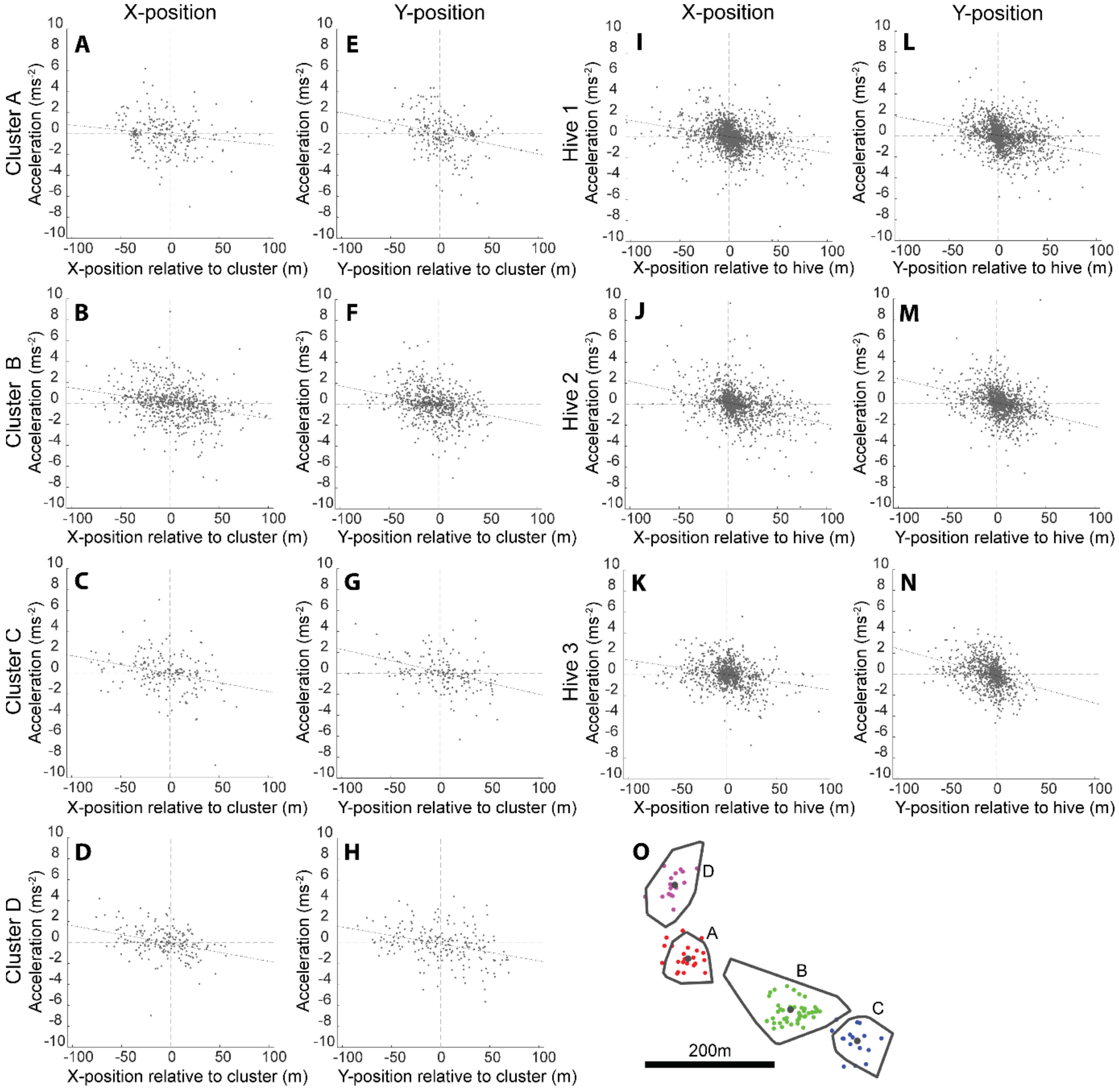

Figure S4. Mean acceleration as a function of position relative to the centres of congregation areas or hives, related to Figure 5

A-D) Distance from the centre of each congregation area in the x-direction (east-west) plotted against the x-component of acceleration in the x-direction. Grey dotted lines in each panel show the regression lines for each distribution.

E-H) Distance from the centre of each congregation area in the y-direction (north-south) plotted against the y-component of acceleration in the y-direction.

I-K) Distance from each hive location vs the x-component of acceleration.

L-N) Distance from each hive location vs the y-component of acceleration. There is a statistically significant negative slope to all distributions indicating that the further drones travel from the centre of the congregation or hive, the more strongly they accelerate back toward the centre.

O) Positions of convoluted sections of flight. The centre of mass of each cluster of data points that we identified as a probable congregation area is marked by a grey circle and labelled A-

D. Convex hull polygons containing all data points assigned to each cluster are outlined in grey. The centre of mass of each convoluted section of flight classified as taking place at congregation area A is represented by a red circle; those at area B by green circles; those of area C by blue circles; and those at area D by magenta circles.

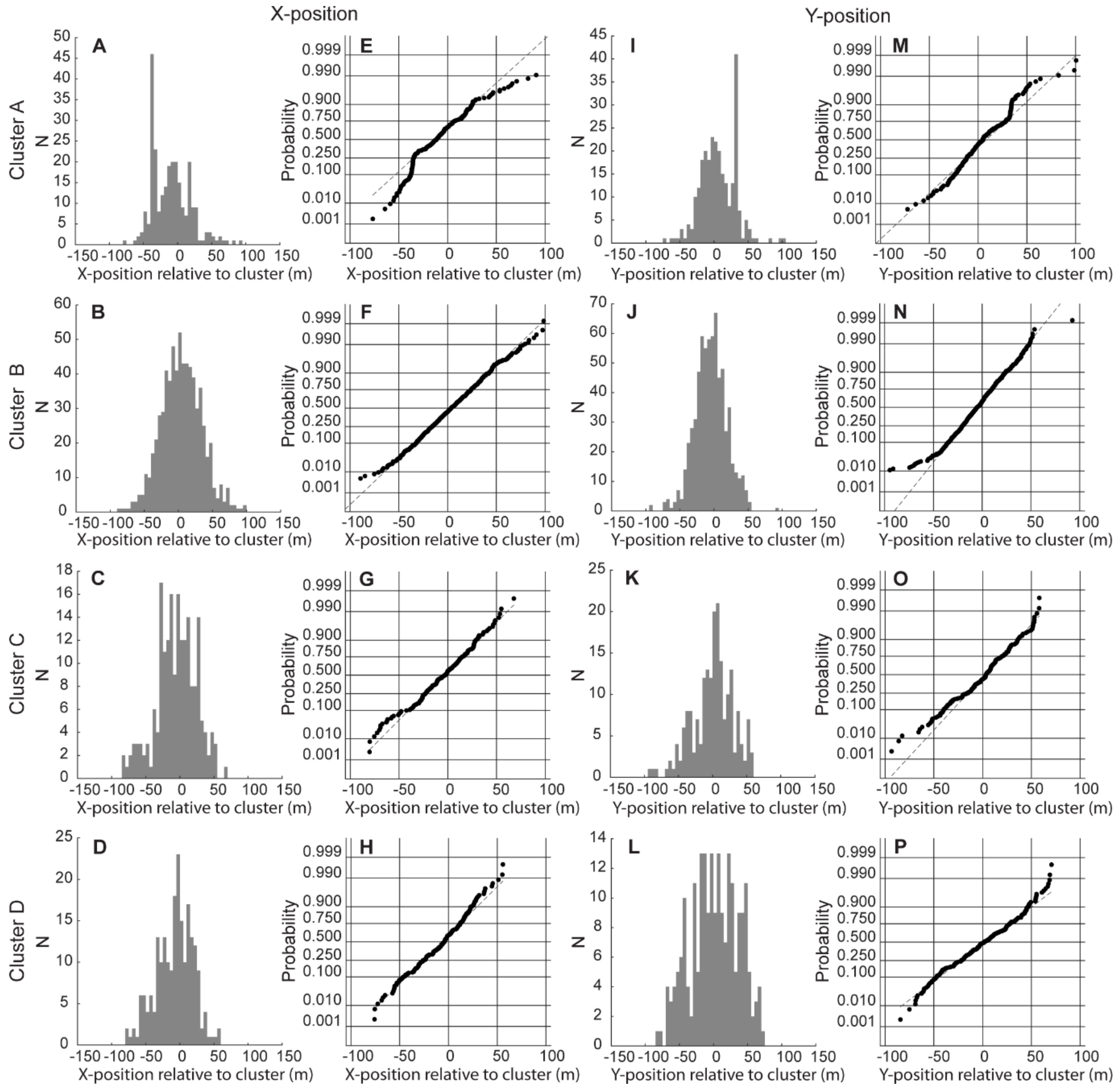

Figure S5. Histograms and normal probability plots showing the distributions of bee position during sections of convoluted flight at four congregation areas, related to Figure 5

A-D) Histograms of the x-position (east-west, relative to cluster centre) of every data point in any convoluted section whose centre of mass was within 50 m of each congregation area centre.

E-H) Normal probability plots for the distributions shown in A-D.

I-L) Histograms of the y-position (north-south, relative to cluster centre) of every data point in any convoluted section whose centre of mass was within 50 m of each congregation area centre.

M-P) Normal probability plots for the distributions shown in I-L. Distributions are approximately Gaussian at their centres, deviating only toward the edges.

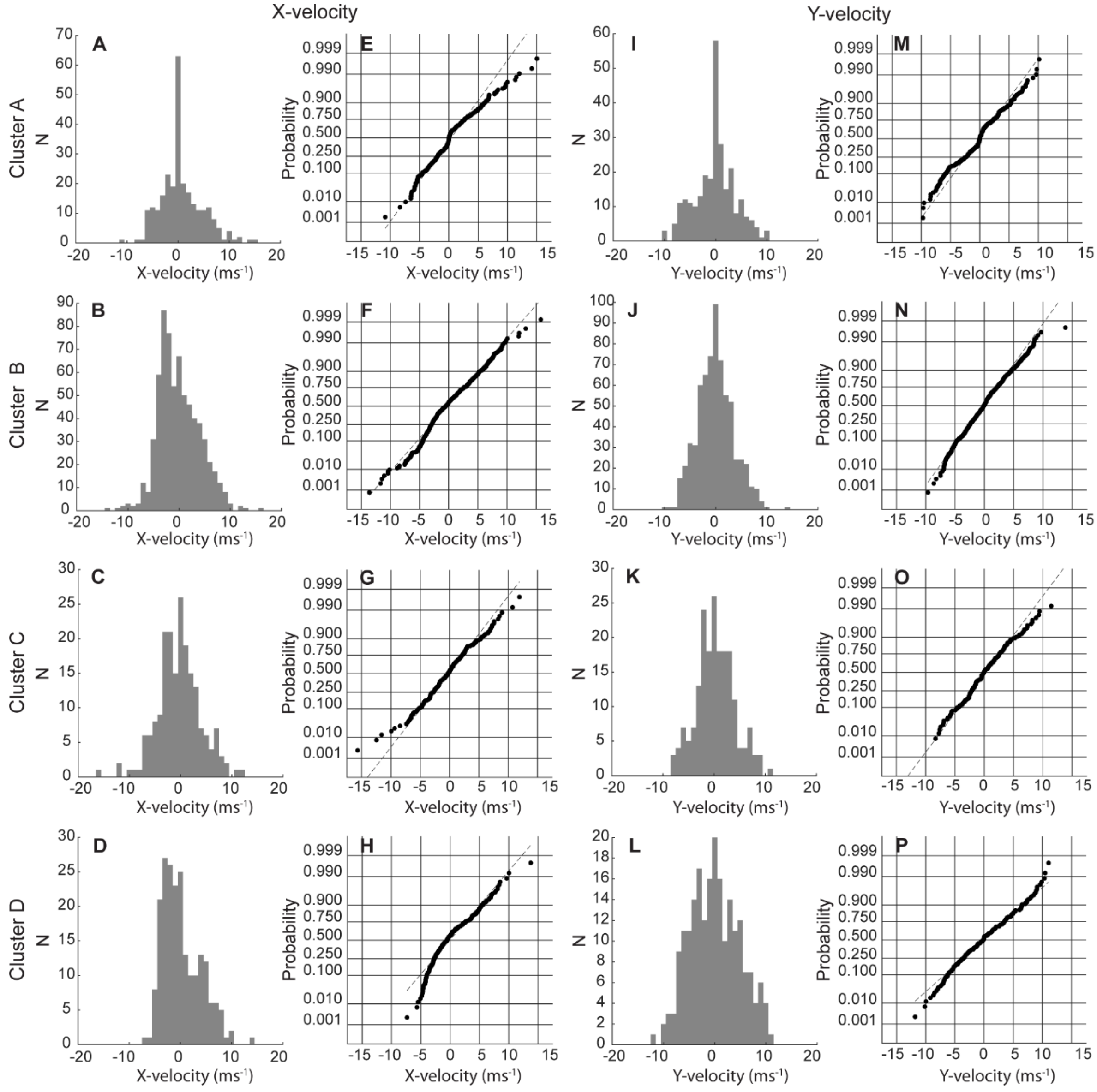

Figure S6. Histograms and normal probability plots showing the distributions of bee velocity during sections of convoluted flight at four congregation areas, related to Figure 5

A-D) Histograms of the x-component of velocity (east-west) of every data point in any convoluted section whose centre of mass was within 50 m of each congregation area centre.

E-H) Normal probability plots for the distributions shown in A-D.

I-L) Histograms of the y-component of velocity (north-south) of every data point in any convoluted section whose centre of mass was within 50 m of each congregation area centre.

M-P) Normal probability plots for the distributions shown in I-L. Distributions are approximately Gaussian at their centres, deviating only toward the edges.

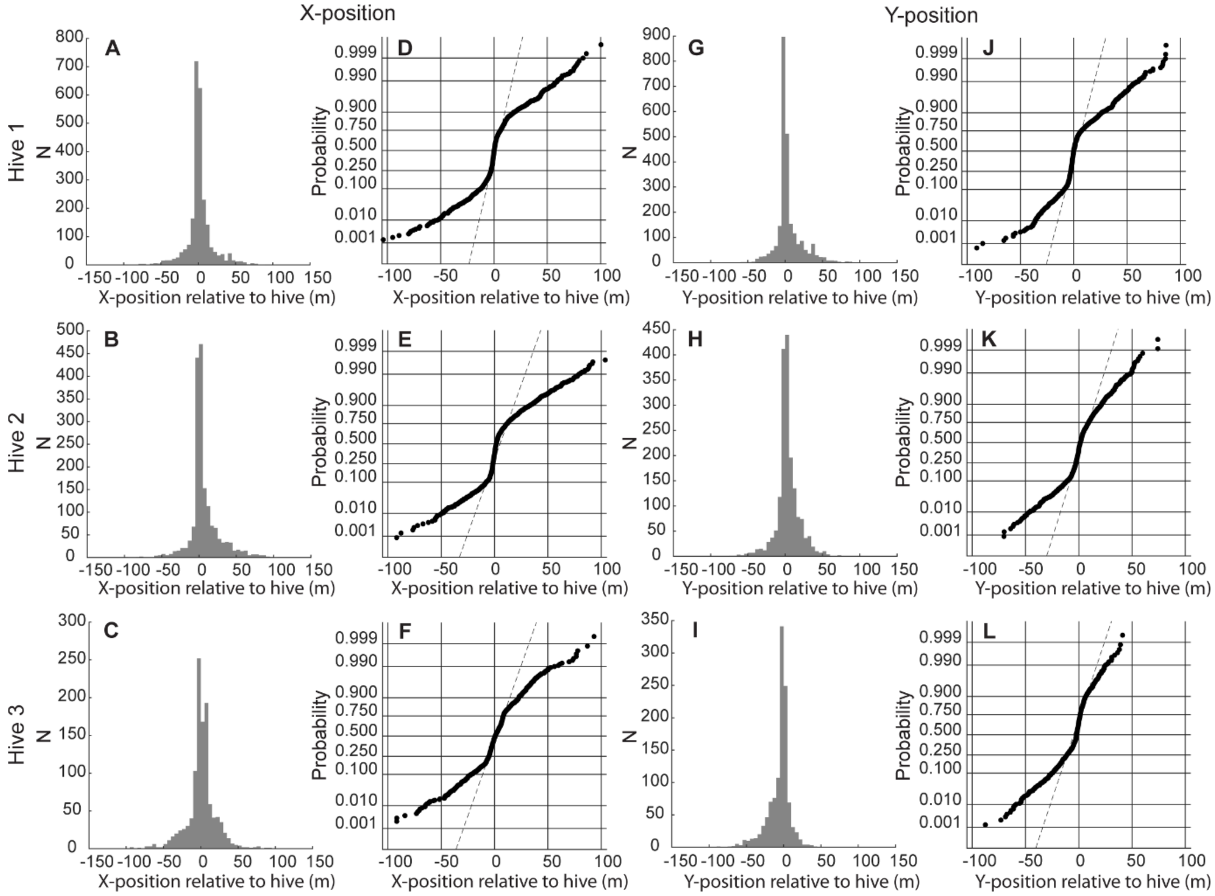

Figure S7. Histograms and normal probability plots showing the distributions of bee position during sections of convoluted flight around three hives, related to Figure 5

A-C) Histograms of the x-position (east-west, relative to hive position) of every data point in any convoluted section whose centre of mass was within 50 m of each hive.

D-F) Normal probability plots for the distributions shown in A-C.

G-I) Histograms of the y-position (north-south, relative to hive position) of every data point in any convoluted section whose centre of mass was within 50 m of each hive.

J-L) Normal probability plots for the distributions shown in G-I. Distributions are narrower than those at swarm locations and fit a Gaussian distribution less well, showing a higher degree of kurtosis.

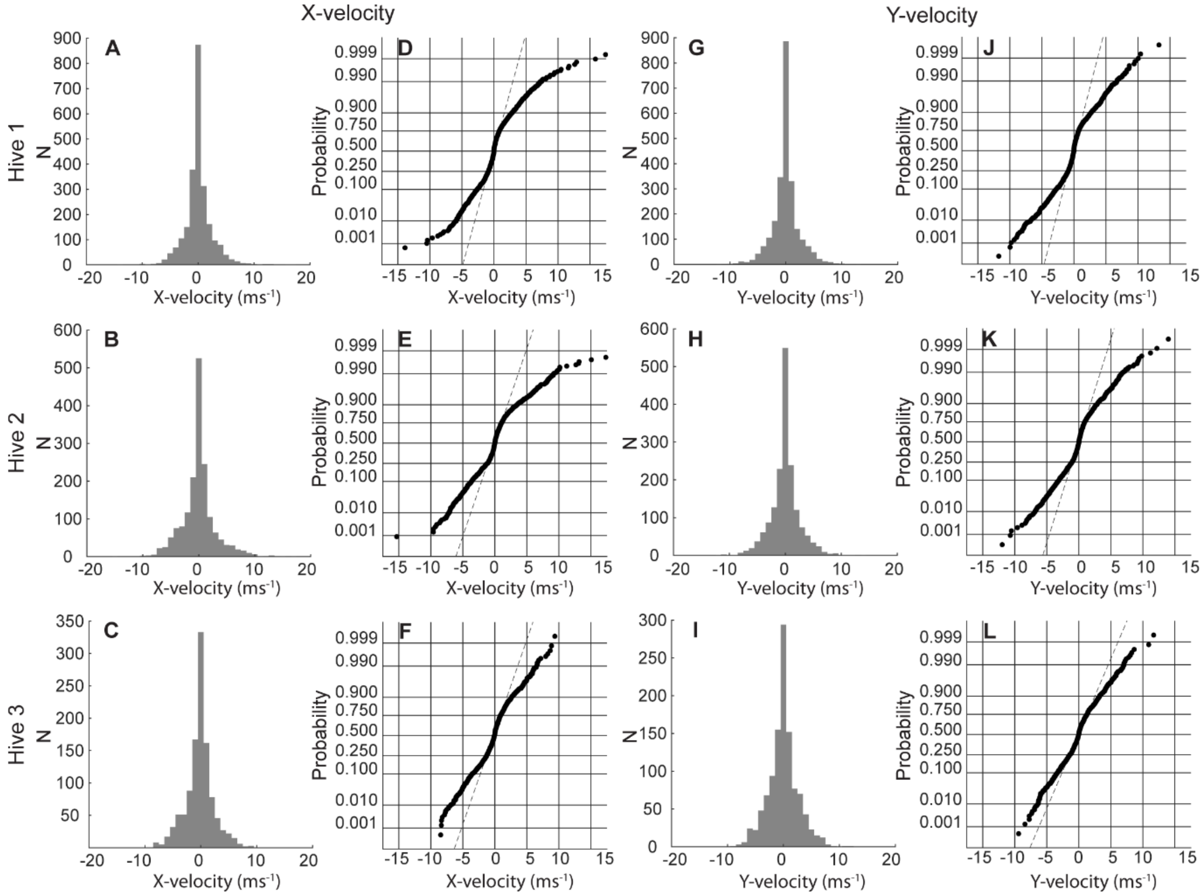

Figure S8. Histograms and normal probability plots showing the distributions of bee velocity during sections of convoluted flight around three hives, related to Figure 5

A-C) Histograms of the x-component of velocity (east-west) of every data point in any convoluted section whose centre of mass was within 50 m of each hive.

D-F) Normal probability plots for the distributions shown in A-C.

G-I) Histograms of the y-component of velocity (north-south) of every data point in any convoluted section whose centre of mass was within 50 m of each hive.

J-L) Normal probability plots for the distributions shown in G-I. Distributions are narrower than those swarm locations and may fit a Gaussian distribution less well.

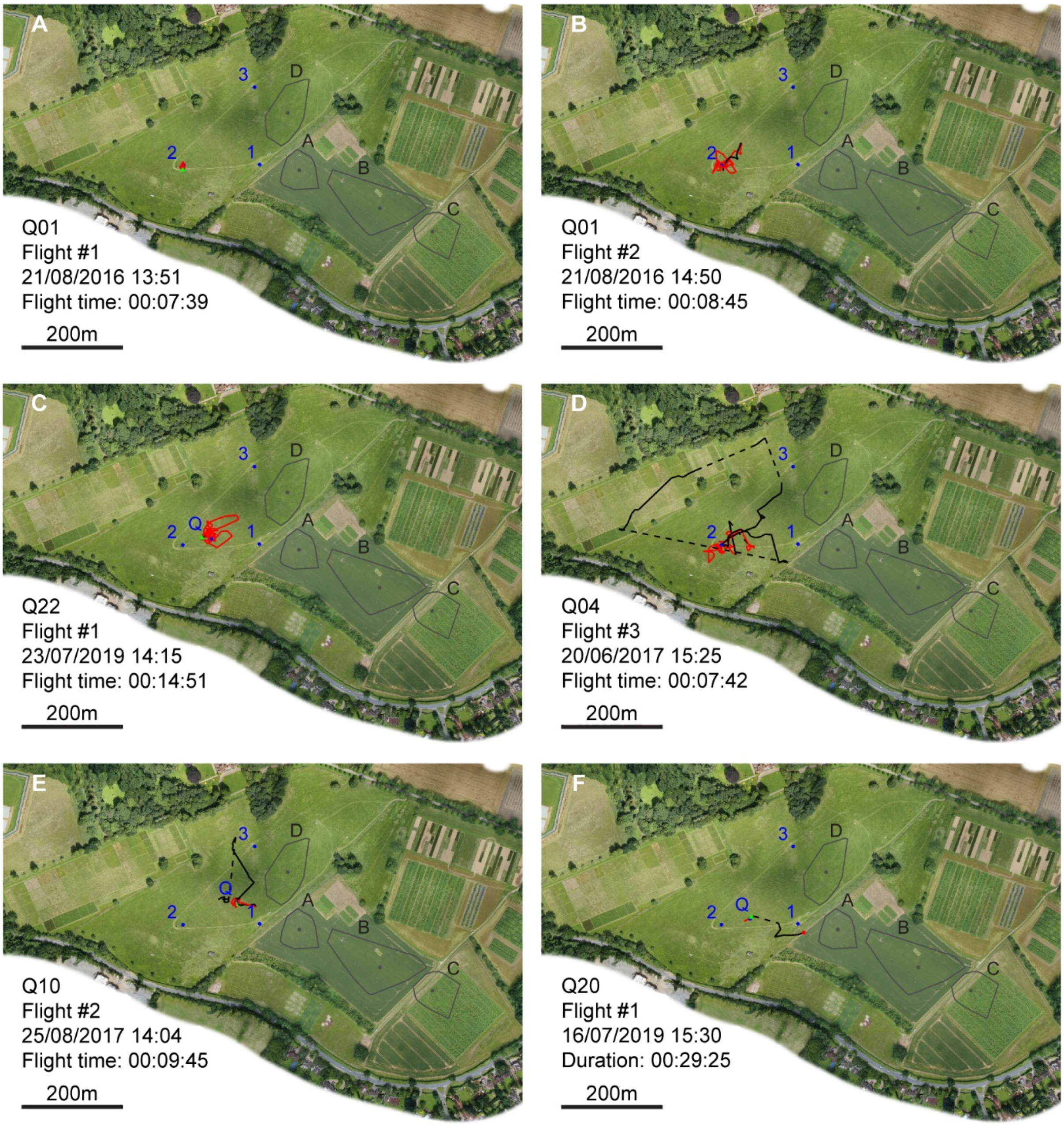

Figure S9. Example flight paths showing first and subsequent flights of virgin queens, related to Figures 3, 4

A) First flight ever undertaken by queen #01. Sections of flight classified as straight are depicted in black; sections of flight classified as convoluted are shown by red lines. Gaps of greater than 30 s between consecutive data points are indicated by dashed lines. The start of the track is marked by a green triangle and the end by a red rectangle. Hives are marked by blue circles and numbered. The centre of mass of each cluster of data points that we identified as a probable drone congregation area is marked by a grey circle and labelled A-D. Convex hull polygons containing all data points assigned to each cluster are outlined in grey. It was common for first flights to remain within 10 m of the hive entrance. Queens typically spent long periods sitting on the ground near the hive, so the time actually spent in flight is given, rather than the overall time spent outside the hive.

B) Second flight undertaken by the same queen, #01, during the same day as her first flight. The bee largely made loops in a very restricted area near the hive entrance, with occasional larger loops, centred on the hive.

C) First flight of queen #22. Queens were kept in mating nuclei at the location marked with a blue circle and labelled ‘Q’.

D) Third flight of queen #04, showing longer range looping flight.

E) Second flight of queen #10, showing flight to the north, during which the queen appears to have mated.

F) First flight of queen #20. The bee lost its transponder and the flight is incomplete, but it returned having mated. The transponder was lost after 3 m 20 s, so it was impossible to calculate the true time spent in flight. The total duration spent outside the hive is given instead.

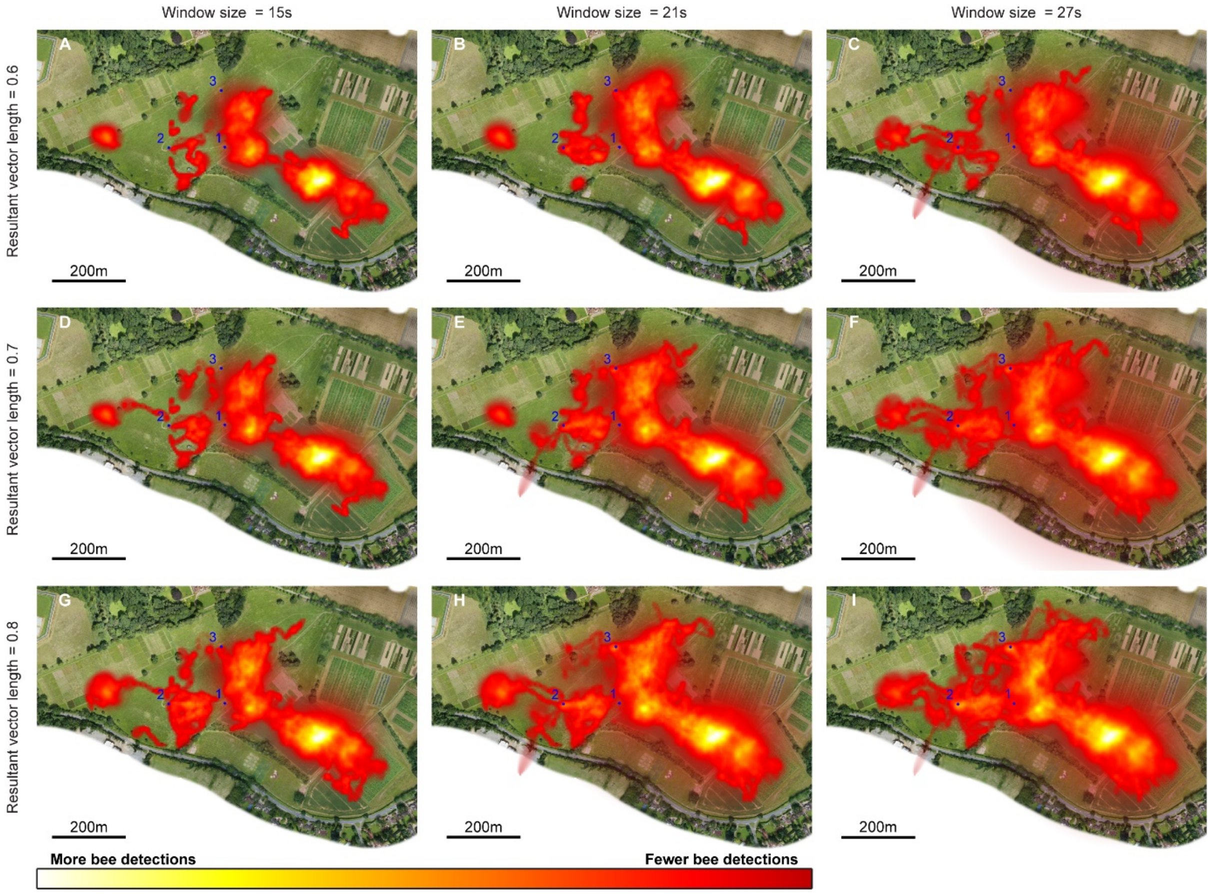

Figure S10. Heat maps demonstrating that detection of convoluted flight is robust to variation in the parameters used, related to Figures 1, S1, STAR methods

A-I) Heat maps showing all convoluted sections of flight recorded in 2016-2017, whose centre of mass was greater than 50 m from all active hives. Hive locations are marked by blue circles and numbered. Areas with brighter, yellower colouration were more visited by drones. Each panel shows the sections of convoluted flight detected by our algorithm when a different combination of two parameters was used (the duration of the moving window over which straightness of the track was calculated, and the threshold minimum vector length used to differentiate straight from convoluted flight). In general, shorter windows or smaller resultant vector lengths result in fewer data points being classified as belonging to convoluted flight, while longer windows or larger vector lengths result in more data points being classified as convoluted flight. In practice, the same sections of flight are typically identified, with data points added to or removed from the start and end of these periods of convoluted flight as the parameters change. In the aggregate, while the boundaries of the regions visited by convoluted flight are changeable, depending on the exact combinations of parameters used, they expand and contract around four constant hotspots, corresponding to the four congregation areas identified in the main text. N = panel A, 57 tracks; B, 79 tracks; C, 113 tracks; D, 79 tracks; E, 111 tracks; F, 146 tracks; G, 92 tracks; H, 135 tracks; I, 165 tracks.

Supplemental tables:

Table S1. Properties of congregation areas and hives, related to Figures 1, 5, 6

Location	GPS	Years	Hives contributing	No. convoluted sections	No. flights	Duration (s) mean ± S.E.	Speed (ms^{-1}) mean ± S.E.
**Cluster A**	51˚ 48.1861' N, 0˚ 22.0368' W	2016, 2017	1,2,3	22	20	133.2 ± 86.1	5.31 ± 0.30
**Cluster B**	51˚ 48.1437' N, 0˚ 21.8994' W	2016, 2017	1,2,3	43	34	115.5 ± 33.1	4.75 ± 0.17
**Cluster C**	51˚ 48.1177' N, 0˚ 21.8097' W	2016, 2017	1,2,3	16	14	70.8 ± 12.1	5.05 ± 0.48
**Cluster D**	51˚ 48.2474' N, 0˚ 22.0546' W	2016, 2017	1,2,3	16	16	110.7± 42.5	5.48 ± 0.25
**Hive 1**	51˚ 48.1920' N, 0˚ 22.1050' W	2016, 2017	1,2,3	107	94	163.2 ± 37.4	2.95 ± 0.16
**Hive 2**	51˚ 48.1909' N, 0˚ 22.2370' W	2016, 2017	2,3	108	88	109.8 ± 17.9	3.13 ± 0.17
**Hive 3**	51˚ 48.2746' N, 0˚ 22.1136' W	2016, 2017	2,3	63	48	159.4 ± 75.4	3.00 ± 0.17

GPS gives the coordinates of the centre of each congregation area in degrees and minutes, calculated as the mean coordinates of all data points in any convoluted section of flight that were assigned to each cluster by Matlab's *clusterdata* function. For the three hive sites, GPS is the location of that hive as recorded by a GPS device. *Years* lists the years in which convoluted sections of flight were found to occur within 50 m of each congregation or hive; *Hives* contributing lists every hive from which at least one drone performing convoluted flight within 50 m of a congregation or hive originated. *No. convoluted sections* and *No. flights* are the total number of convoluted sections of flight occurring at each location and the number of different flight segments those convoluted sections were part of. *Duration* and *Speed* are, respectively, the mean duration and speed of each convoluted section of flight whose centre of mass was within 50 m of each location.

Table S2: Results of regressions of position vs acceleration for four congregation areas and three hives, related to Figure 5

Location	Direction	Slope	Y-intercept	X-intercept	R^2	F	d.f.	P
**Cluster A**	X	-0.009	-0.13	-13.19	0.03	7.76	1, 257	**0.0058**
	Y	-0.020	0.05	2.33	0.09	23.99	1, 257	**<0.0001**
**Cluster B**	X	-0.015	0.05	3.39	0.07	53.40	1, 661	**<0.0001**
	Y	-0.019	-0.10	-5.53	0.10	73.98	1, 661	**<0.0001**
**Cluster C**	X	-0.017	-0.06	-3.22	0.08	16.74	1, 193	**0.0001**
	Y	-0.021	0.16	7.31	0.11	24.30	1, 193	**<0.0001**
**Cluster D**	X	-0.017	-0.10	-5.58	0.11	25.89	1, 203	**<0.0001**
	Y	-0.016	-0.10	-6.19	0.11	26.29	1, 203	**<0.0001**
**Hive 1**	X	-0.015	0.03	1.83	0.07	172.00	1, 2354	**<0.0001**
	Y	-0.018	0.05	3.12	0.09	218.86	1, 2354	**<0.0001**
**Hive 2**	X	-0.021	0.15	7.09	0.09	166.82	1, 1642	**<0.0001**
	Y	-0.023	0.08	3.57	0.10	175.07	1, 1642	**<0.0001**
**Hive 3**	X	-0.014	0.00	0.16	0.07	75.64	1, 1079	**<0.0001**
	Y	-0.026	-0.16	-6.20	0.12	147.12	1, 1079	**<0.0001**

For each location there were two regressions: one for the x-position vs x-component of acceleration and one for the y-position vs y-component of acceleration; these are indicated by the code in the *Direction* column. *Slope* and *Y-intercept* are the properties defining the regression line through each distribution. *X-intercept* is the location, relative to the centre of the cluster or hive, at which acceleration is zero. The distributions and regression lines are all shown in Figure S4. Columns *F*, *d.f.* and *P* give the test statistic, degrees of freedom and P-value of a linear regression on each distribution. Tests that were significant at a 0.05 level are indicated in bold type.
